# SWATH-MS Quantitative Analysis of Proteins in the Rice Inferior and Superior Spikelets during Grain Filling

**DOI:** 10.3389/fpls.2016.01926

**Published:** 2016-12-20

**Authors:** Fu-Yuan Zhu, Mo-Xian Chen, Yu-Wen Su, Xuezhong Xu, Neng-Hui Ye, Yun-Ying Cao, Sheng Lin, Tie-Yuan Liu, Hao-Xuan Li, Guan-Qun Wang, Yu Jin, Yong-Hai Gu, Wai-Lung Chan, Clive Lo, Xinxiang Peng, Guohui Zhu, Jianhua Zhang

**Affiliations:** ^1^College of Life Sciences, South China Agricultural UniversityGuangzhou, China; ^2^State Key Laboratory of Agrobiotechnology, School of Life Sciences, The Chinese University of Hong KongHong Kong, Hong Kong; ^3^Shenzhen Research Institute, The Chinese University of Hong KongShenzhen, China; ^4^School of Pharmacy, Nanjing Medical UniversityNanjing, China; ^5^College of Life Sciences, Nantong UniversityNantong, China; ^6^College of Life Sciences, Fujian Agriculture and Forestry UniversityFuzhou, China; ^7^The Rice Research Institute, Guangdong Academy of Agricultural SciencesGuangzhou, China; ^8^School of Biological Science, The University of Hong KongHong Kong, China

**Keywords:** SWATH-MS analysis, grain filling, post-transcriptional regulation, proteomics, rice

## Abstract

Modern rice cultivars have large panicle but their yield potential is often not fully achieved due to poor grain-filling of late-flowering inferior spikelets (IS). Our earlier work suggested a broad transcriptional reprogramming during grain filling and showed a difference in gene expression between IS and earlier-flowering superior spikelets (SS). However, the links between the abundances of transcripts and their corresponding proteins are unclear. In this study, a SWATH-MS (sequential window acquisition of all theoretical spectra-mass spectrometry) -based quantitative proteomic analysis has been applied to investigate SS and IS proteomes. A total of 304 proteins of widely differing functionality were observed to be differentially expressed between IS and SS. Detailed gene ontology analysis indicated that several biological processes including photosynthesis, protein metabolism, and energy metabolism are differentially regulated. Further correlation analysis revealed that abundances of most of the differentially expressed proteins are not correlated to the respective transcript levels, indicating that an extra layer of gene regulation which may exist during rice grain filling. Our findings raised an intriguing possibility that these candidate proteins may be crucial in determining the poor grain-filling of IS. Therefore, we hypothesize that the regulation of proteome changes not only occurs at the transcriptional, but also at the post-transcriptional level, during grain filling in rice.

## Introduction

Rice (*Oryza sativa* L.), one of the most important food crops worldwide, is now consumed by more than 3 billion people and provides 35–60% of the dietary calories. Two major breakthroughs were witnesses in past 50 years to break yield barrier in rice. The first one occurred during the First Green Revolution in 1960s with the introduction of semi-dwarf gene. The second breakthrough was made in the early 1970s in China by developing hybrid rice (Zhang, 2011). China has the responsibility to feed 20% of the global population with only about 5% of the water resources and 7% of the world’s arable land. Despite limited natural resources, however, China’s grain production achieved a 4.5-fold increase from the 1950s to early 1990s, whereas the national population increased by 2.5-fold and nearly half of the cropping lands were vanished during this period (Zhang, 2011). Thus, further increase in yield is the major challenge to meet the demands of growing food requirement in future. To increase rice yield based on optimizing growth conditions, water and nutrient supply has already been exploited. Introducing new strategies based on modern molecular biology and plant engineering is necessary to achieve another boost of grain yield. Over the last 15 years, concerted efforts involving China and international programs have aimed to develop ‘super hybrid rice’ for breaking the yield ceiling (Yang and Zhang, 2010).

The ‘super’ hybrid rice cultivars have large number of spikelets per panicle. They offer 8–20% increase in yield potential in comparison to conventional rice cultivars such as 93-11 (YD6). However, the yield potential of these ‘super’ hybrid rice cultivars has not been fully achieved due to several drawbacks. First, they tend to have lower percentage of filled spikelets in a panicle. Approximately 79% spikelets filling is observed for super rice compared to that of conventional rice (∼89%) (Yang and Zhang, 2010). Second, the yield is often unstable due to large variations noted by cropping year and location. Third, high percentage of unfilled spikelets (5–10%) has been observed in super hybrid cultivars in comparison to conventional varieties (2–5%). The late-flowered inferior spikelets (IS) of super rice are filled slowly and in many cases their filling is aborted. Such poorly developed grains in the spikelets are undesired trait for crops. In rice, the classification of superior and IS is according to their flowering date and position within a panicle. Superior spikelets (SS) usually are present on apical primary branches. The early flowering and fast grain filling develop these spikelets into larger and heavier grains. In contrast, IS present on proximal secondary branches, flower later, fill slowly and have smaller and lighter grains (Yang and Zhang, 2010). Commonly, rice yield is determined by three factors including grain number per panicle, panicle number per unit area, and grain weight (Huang et al., 2013). The first two factors have been optimized in the super rice variety. Grain size (length, width, and thickness) and degree of filling are two parameters, which can determine the grain weight. In the past decade, considerable efforts have been made to dissect the key factors involved in controlling rice grain weight (Zhou et al., 2013). A few genes controlling grain size have been documented mainly from map-based cloning. For example, mutation of rice heterotrimeric G protein alpha subunit (*RGA1*) has been proposed to reduce seed size (Ashikari et al., 1999). One RING-type E3 ubiquitin ligase, cloned from *GRAIN WIDTH2*, a major QTL for rice grain weight and width, has been observed to inhibit cell division by degrading its related components (Song et al., 2007). The QTL *GRAIN SIZE 3* encodes a large protein containing several domains which function in determining grain length and thickness (Mao et al., 2010). Notably, only one gene has been reported to influence grain filling. The mutant of this locus, named as *grain incomplete filling1* (*gif1*), produced chalky grains as resulted from incomplete grain filling (Wang et al., 2008). This gene encodes a cell wall invertase, which participates in carbon partitioning during early grain filling. Previously, our group focused on developing field management practices to increase grain filling in super hybrid rice cultivars. Using physiological, biochemical, and molecular approaches, our earlier works revealed that the variable and low grain filling rate is the major cause for the variable yield performance of super rice. Several factors have been suggested to be involved in the regulation of rice grain filling. For example, moderate water stress (Yang et al., 2001), the balance of several plant hormones (i.e., cytokinins, abscisic acid, and ethylene) in spikelets (Yang et al., 2003, 2006; Zhang et al., 2009), the activities of enzymes involved in starch metabolism (Zhu et al., 2011), the polyamine content (Yang et al., 2007) and the carbon remobilization from the pre-stored reserve (Fu et al., 2011). However, the molecular regulatory mechanisms underlying rice grain filling is still largely unknown.

Few proteomic investigations on differential grain-filling between rice IS and SS were carried out previously using 2D gel-based approaches (Zhang et al., 2014; Chen et al., 2016). However, this approach does not provide accurate quantification and large-scale identification of differentially expressed proteins (Zhang et al., 2014; Chen et al., 2016). In this study, the changes in the proteome profile between rice IS and SS during the grain-filling process were examined using the recently developed SWATH-MS (Sequential Window Acquisition of All Theoretical Mass Spectra -Mass Spectrometry)-based quantitative proteomics approach in combination with bioinformatic analysis. SWATH-MS is a data-independent acquisition (DIA) method. The data was acquired by repeatedly cycling through sequential mass windows over the whole chromatographic elution range generating a complete recording of all analytes in the sample. This approach collects data on the low intensity commonly missed during data-dependent acquisition (DDA) methods leading to incomplete identification and quantification (Bourassa et al., 2015). Meanwhile, SWATH-MS can provide high quantification accuracy, dynamic range, as well as the reproducibility of selected reaction monitoring (SRM) (Gillet et al., 2012; Liu et al., 2014). Therefore, SWATH-MS has been considered to be a more promising and popular quantitative approach for protein identification and verification (Gillet et al., 2012; Liu et al., 2013, 2014) in comparison to the conventional gel-based or label-based proteomics approach such as 2D and iTRAQ. It has been extensively applied to detect global proteome changes in microbial, human, and animal systems (Liu et al., 2013, 2014; Haverland et al., 2014). Here, we revealed a total of 1853 proteins which were quantitatively identified by SWATH-MS in rice spikelets, 304 of which were found to be differentially expressed during the grain-filling between IS and SS (at *P* < 0.05). Alterations were observed in different biological processes including photosynthetic process, protein and energy metabolism as well as the sucrose to starch conversion process. Detailed bioinformatics data analysis will enable substantial information on the proteome changes between the IS and SS to be obtained, offering deeper insights into the molecular mechanisms of grain filling in rice. In summary, our SWATH-MS based proteomic study can provide novel insights into the control mechanism of rice grain filling at the protein levels and find out crucial regulators for potential application to increase rice yield in future.

## Materials and Methods

### Plant Materials and Sampling

Experiments were conducted at research farm of South China Agricultural University during the rice growing season. A large-panicle rice cultivar, YD-6 (Yangdao 6, an indica inbred cultivar) was used for the study. The germinated seeds were grown in the paddy field and seedlings were transplanted into the soil containing N-P-K at 100, 40, and 70 mg kg^-1^, respectively, with suitable hill spacing. Approximately two hundred panicles flowered on the same day were tagged and sampled. The flowering date and position of each spikelet on the tagged panicles were recorded. The SS and IS were distinguished based on the previous report (Ishimaru et al., 2003). A total of 80 tagged panicles were sampled at 9 days after flowering, immediately frozen in liquid nitrogen and stored in -80°C for further analysis.

### Protein Extraction

The IS and SS samples (approximately 1 g) were ground in liquid nitrogen and homogenized with 10x volume of trichloroacetic acid (TCA)/Acetone. The homogenates were centrifuged in a JA-12 rotor (Beckman) at 16000 *g* at 4°C for 5 min and the supernatant was carefully removed. Protein pellets were suspended in 10x volume of 80% MeOH/0.1 M NH_4_OAc, followed by the centrifugation at 16000 *g* for 5 min at 4°C. The pellets were re-suspended in 10x volume of 80% acetone, followed by another centrifugation. Subsequently, the protein pellets were dissolved in 8 mL SDT buffer (4% SDS, 0.1 M DTT, and 0.1 M MOPS/HCl, pH 8.0) and the homogenized samples were centrifuged at 15000 rpm for 2 min. Then they were incubated at 95°C for 5–10 min. After centrifugation for two times at 4°C, the supernatants were collected and added with 4x volume of chilled 80% acetone for overnight participation at -20°C. Proteins were then centrifuged at 4000 rpm for 15 min and pellets were washed with 10x volume of 80% acetone. After centrifugation, the pellets were air-dried. Finally, the protein pellet was dissolved with 1mL urea buffer (6 M urea in 200 mM MOPS-Cl/4 mM CaCl_2_, pH 8.0) by sonication (10 s on, 6 s off; 20 cycles). Protein concentrations were accurately estimated by the Bradford method (Bio-Rad). Seven biological replicates were prepared from IS and SS.

Protein samples (100 μg each) were reduced by 10 mM DTT at 50°C for 30–40 min with gentle shaking and then alkylated at room temperature for 40 min by iodoacetamide (IAA) to a final concentration of 40 mM. Samples were digested by trypsin (1 μg trypsin for 50 μg protein) and incubated at 37°C overnight. Subsequently, peptides from different proteins samples were acidified with 10% trifluoroacetic acid and desalted by SepPak C18 cartridges (Waters) following the manufacturer’s instructions and dried in speed vacuum concentrator. Peptide samples were either stored at -80°C if not analyzed immediately or dissolved in 0.1% formic acid for LC-MS/MS analysis.

### SWATH-MS Measurement and Analysis

All samples were analyzed using the Eksigent NanoLC-2DPlus system coupled with the cHiPLC nanoflex system in Trap-Elute mode by the Triple TOF 5600 mass spectrometer (SCIEX, USA) as previously described (Zhu et al., 2016). Briefly, peptide samples were first loaded on a cHiPLC trap (3 μm, ChromXP C18CL, 120 Å, 0.5 mm × 200 μm) with 95% water (0.1% formic acid) and 5% acetonitrile (0.1% formic acid) at 500 nL/min for 15-min. Subsequently, the samples were separated on a cHiPLC column (3 μm, ChromXP C18CL, 120 Å, 15 cm × 75 μm) in an elution gradient of 5–35% acetonitrile at 300 nL/min for 120-min. Trap and column were maintained at 30°C for retention time stability. The eluent from the column was analyzed by the Triple TOF 5600 mass spectrometer in positive ion mode with a nano-ion spray voltage of 2300 V. Data-dependent acquisition (DDA) method was first performed to generate the SWATH-MS spectral library. For the detailed experiment, a survey scan of 250 ms (TOF-MS) in the range 350–1250 m/z was performed to collect the MS1 spectra and the top 40 precursor ions with charge state from +2 to +5 was selected for subsequent fragmentation with an accumulation time of 50 ms per MS/MS experiment for a total cycle time of 2.3 s and MS/MS spectra was acquired in the range 100–1800 m/z.

In the SWATH analysis, the same peptide samples were subjected to cyclic DIA of mass spectra in a similar manner to above established methods. The mass spectrometer was operated on a 50 ms survey scan and all precursors were subject to fragmentation. All DDA mass spectrometry files were searched to generate a reference spectral library using the ProteinPilot software v4.5 (SCIEX) with the Paragon algorithm. The searching was conducted against the UniProt Swiss-Prot *O. sativa* protein database (July, 2015). The output of this search is a group file, which contains the following information that is required for spectral alignment and targeted data extraction of DIA: protein name, UniProt accession, peptide sequence, precursor charge and fragment ions, relative intensity, and retention time, etc. Subsequently, the acquired targeted data extraction of DIA samples was loaded into the PeakView software v1.2 (SCIEX) under the reference spectral library. All loaded DIA files were exported into.txt format using an extraction window of 10 min with the following parameters: ion library mass tolerance (50 ppm); eight peptides; five transitions; peptide confidence of >99%; exclusion of shared peptides. The quantified proteins and associated peptides were further displayed and exported to MarkerView (SCIEX) format in the generation of three distinct files containing the quantitative output for (1) extracted peak area for individual fragment ion; (2) sum intensity of fragment ion areas for a given peptide; (3) sum intensity of peptide areas for a given protein. With the normalization of the total areas of seven biological replicates, relative quantitation for proteins (fold change) during the grain-filling in the IS and SS was obtained and analyzed by *t*-test (MarkerView) (**Supplementary Table S2**). Proteins with a fold change of >1.2 or <0.8 (*P*-value < 0.05) were considered as differentially expressed proteins in this study. All the raw data have been submitted into the PRIDE PRoteomics IDEntifications (PRIDE) database with the accession number PXD005401.

### Quantitative Real-Time RT-PCR Analysis

Transcriptional analysis of genes corresponding to differentially expressed proteins (**Supplementary Table S1**) was performed by quantitative real-time RT-PCR. RNA samples were prepared from IS and SS at 9-days after fertilization_._ Total RNA was reverse-transcribed into cDNA using M-MLV reverse transcriptase (Promega) according to the protocol supplied by the manufacturer. The SYBR Green Mix (Applied Biosystems) was used as the PCR reagent. PCR was conducted on a StepOne Plus realtime PCR system using the program: 95°C for 10 min followed by 40 cycles of 95°C for 15 s and 56°C for 1 min. Calculation for fold changes in expression level was performed according to the comparative CT value method (Schmittgen and Livak, 2008).

## Results

### Selection of Appropriate Sampling Time Point Using Physiological Characterization

**FIGURE 1:**
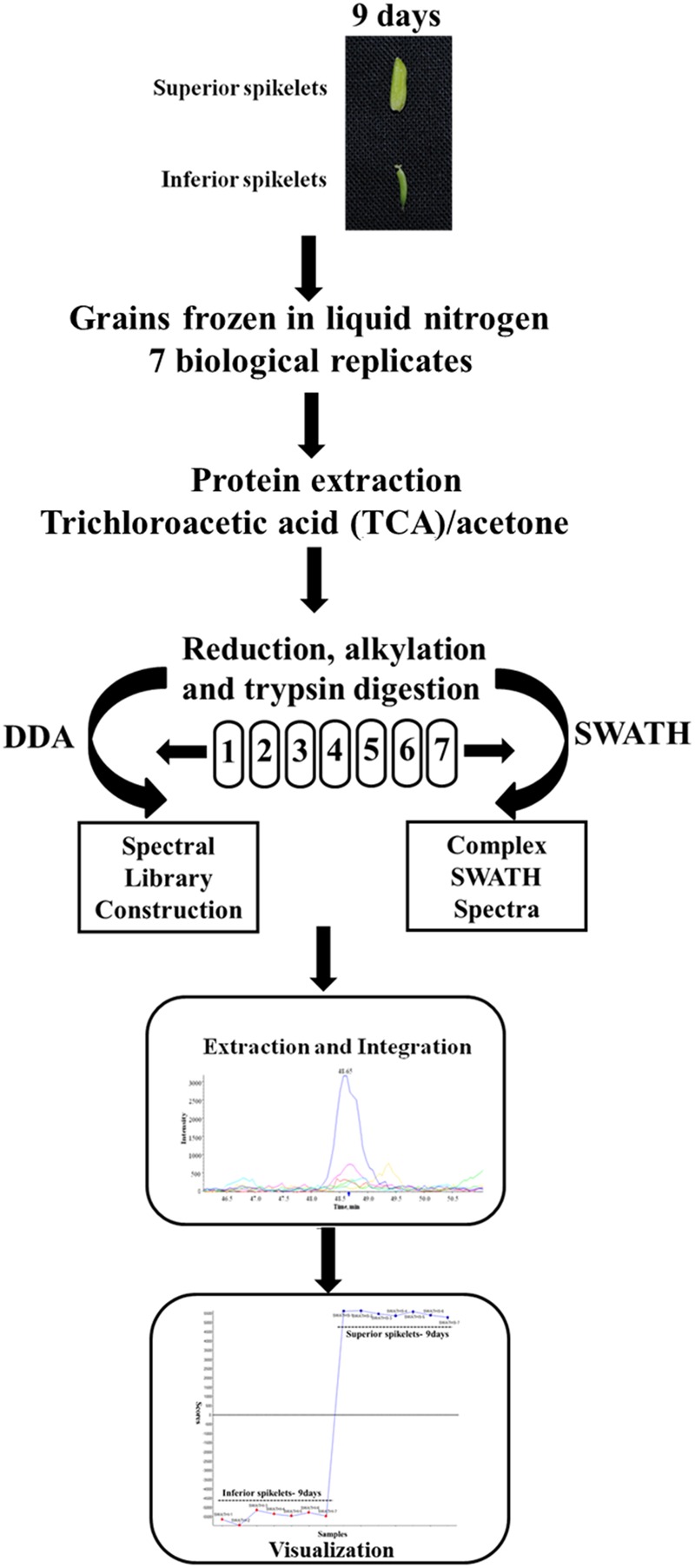
**Workflow for the SWATH-based quantitative proteomic experiment.** Protein samples were obtained from rice inferior spikelets (IS) and superior spikelets (SS) at 9 days after flowering (DAF). Seven biological replicates of IS and SS samples were digested and desalted. Each peptides sample was injected separately in a data-dependent acquisition (DDA) mode for the spectral library construction and in a SWATH mode. The spectral library from DDA runs was used by the Peakview and Markerview to extract the peptide and the quantification information on each of the SWATH runs.

In the present study, different grain-filling processes were observed in SS and IS of rice (Yangdao 6). SS showed rapid increase grain weight and elongation after flowering, whereas IS hardly elongated and grain weight increased relatively slowly. We previously demonstrated that the maximum grain-filling rate differences occurred at 9 days after flowering (DAF) for SS and IS (Zhu et al., 2011). Therefore, the 9 DAF of SS and IS were used in this proteome study and to investigate the differentially expressed proteins between superior and IS during grain filling by the SWATH-MS method. Tryptic and desalted peptides samples of proteins extracted from SS and IS were injected on the TripleTOF 5600 in DDA (Data dependent Acquisition) mode for the generation of reference MS-spectral library. Each sample containing seven biological replicates was used for SWATH-MS analysis. The schematic workflow of the experimental design is shown in **Figure 1**.

### Comprehensive Inventory of Proteome Changes between IS and SS

To comprehensively evaluate the proteome changes between the superior and IS during grain-filling processes, comparative proteomic analysis was performed. A spectral library of 2026 proteins was generated with FDR < 1%. The library and the SWATH-MS data were uploaded into the PeakView software for peak area integration of the identified peptides. Relative quantitation (fold changes) and statistical analyses (*t*-tests) was subsequently conducted using MarkerView software. For the differentially expressed proteins between IS and SS, only those showing the fold changes with *p*-value below 0.05 were considered. Data from this study revealed a total of 304 proteins showing significant changes in protein abundance, which included 241 decreased and 63 increased proteins in the IS compared to SS. To examine the potential coordinately regulated proteins contributing to grain-filling processes, the differentially expressed proteins were assigned to 26 functional categories according to the MapMAN BIN system^1^ (**Supplementary Table S1**).

**FIGURE 2:**
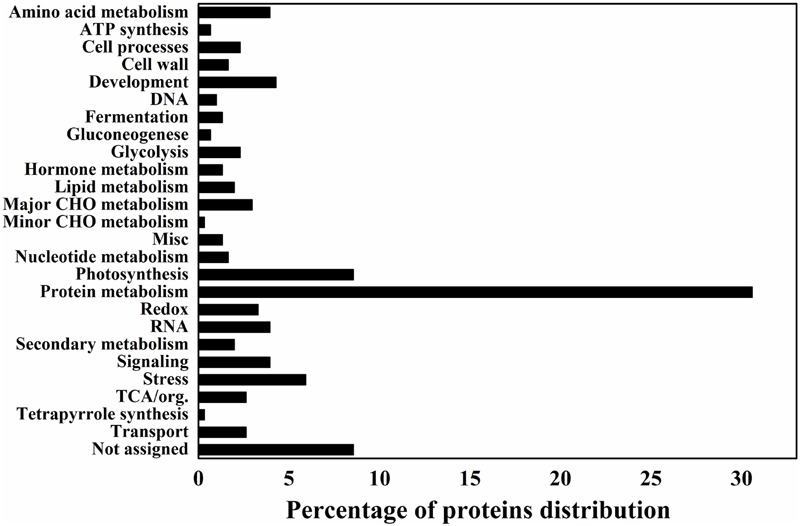
**Differentially expressed proteins distribution by the MapMAN BIN system.** The plots reveal the differentially expressed proteins distribution of 26 functional categories by the MapMAN BIN system.

Data analysis was also performed to categorize the differentially expressed proteins in different functional categories to provide a quick view on the most significant protein changes during the grain-filling between IS and SS. Accordingly, proteins representing the categories “Protein metabolism” and “Photosynthesis” make up the large part of differentially expressed proteins, indicated that they were greatly affected during rice grain-filling and play important roles between IS and SS (**Figure 2**). Interestingly, proteins representing the categories “protein metabolism,” “photosynthesis” and “major CHO metabolism” constituted 30.6, 8.5, and 3% of all protein with decreased abundance (IS vs. SS), respectively, making up a relatively high proportion (**Figure 2**). On the other hand, “development” and “stress” constitute the top functional categories for proteins with increasing abundance (**Figure 2**). Such findings suggested that those biological processes including protein and major CHO metabolism as well as photosynthesis are likely to be negatively affected during rice grain-filling in IS, while the processes of development and stress responses are highly correlated with the poor graining of IS. More interestingly, in this study, the number of proteins with reduced abundances is 3–4 times more than the number of proteins with increased abundances, similar to a previous gel-based proteomics investigation comparing the grain-filling process between IS and SS (Chen et al., 2016). In addition, we analyzed the identified proteins from SWATH-MS data using a multifaceted bioinformatic approach for the comprehensive elaboration of differentially expressed proteins between IS and SS. Gene Ontology (GO) analysis were performed using three main ontologies including biological process, cellular component, and molecular function as shown in Supplementary Figure S1. We found that most of the differentially expressed proteins concentrated into the metabolic processes (Supplementary Figure S1). Functional GO enrichment analysis also revealed that proteins of the nature of RNA-binding or catalytic features with the cytoplasm localization were enriched among differentially expressed proteins, indicating a dominant role of specific cellular localization and biochemistry characteristics during the rice grain-filling (Supplementary Figure S1).

### Slowed Photosynthetic Rate in the Inferior Spikeletes

The typical IS are physically located on the proximal secondary branches in a rice panicle where the grains receive less photosynthesis during the grain-filling processes in comparison to SS (Peng et al., 2014). Consistently, our quantitative proteomics data revealed that most of the differentially expressed photosynthesis-related proteins showed reduced abundance during the grain-filling in IS compared to SS. These proteins are mainly subunits of photosynthetic enzyme complexes including photosystems (PSI and PSII), ATP synthases and RUBISCO (**Supplementary Table S1**), suggesting that the photosynthetic processes were slowed significantly in poor-graining of IS. Six core proteins of PSI-PSII complex and five cholorplastic, mitochrondrial, and cytosolic ATP synthases are important components of photosynthesis, which functions together to transform light energy into chemical energy in plants (Nelson and Yocum, 2006). Five chlorophyll a-b binding proteins (Os03g39610, Os04g38410, Os07g37240, Os08g33820, and Os11g13890) are essential for chlorophyll synthesis in plants (Tanaka and Tanaka, 2011). The overall down-regulation of photosynthesis-related proteins in IS possibly led to an unfavorable environment that in turn affected the activity of enzymes involved in glycolysis and sucrose-to-starch conversion, thus contributing to the poor graining of IS. Moreover, abundances of proteins involved in the photosynthetic photorespiration (Os03g57220 and Os12g22030) and Calvin cycle (e.g., Os03g03720 and Os01g02880) were also shown to be decreased in IS compared to SS, consistent with the photosynthetic proteome changes occurring in previous investigation of proteome changes between IS and SS (Zhang et al., 2014; Chen et al., 2016). However, the number of photosynthesis-related proteins identified in this study were three–fourfold more in comparison to the proteomics data of previous 2D gel-based studies between IS and SS (Zhang et al., 2014, 2015), indicating a high throughput and accuracy advantage of SWATH-based technology used in this study and also enrich the understanding of photosynthesis proteins involve the grain-filling process.

### Down-Regulation of Protein Metabolism in the Inferior Spikeletes Compared to the Superior Spikeletes

The protein metabolism category was also highly represented in the proteome of IS and SS, making up the largest proportion (30.6%) among all the identified differentially expressed proteins. Proteome changes in protein metabolism category included protein biosynthesis, folding and activation as well as degradation. A total of 51 proteins involved in protein synthesis were identified by SWATH-MS analysis in this study, which were substantially higher in terms of number of the translation-related proteins reported previously for the proteomes comparison between IS and SS (Zhang et al., 2015; Chen et al., 2016). Among them, there were 41 ribosomal proteins, which have been linked with cell structure and plant development (Yao et al., 2008), six proteins involved in translation initiation and four proteins involved in elongation showed reduced abundance in IS during the grain-filling (**Supplementary Table S1**), suggesting an suppression of translational activities in the IS development. This may be associated with the reduced abundance of different photosynthetic proteins in IS, as described above. In contrast, several proteins involved in protein modification showed increased abundance in the IS. Generally, protein dysfunction is an inevitable common consequence of low grain-filling rate in rice (Wang et al., 2008), thus it is necessary to require the assistance of modification proteins such as heat-shock protein (Hsp) and proteasomes for proper folding, assembly and degradation (Wang et al., 2004; Kaucsar et al., 2014). Here, one Hsp protein (Os10g32550), one alpha subunit of T-complex 1 chaperonin protein (Os04g46620) as well as one proteasome (Os02g21970) exhibited increased abundance in the IS compared to SS, indicating their potential role in the protein repair to cope with the retardation of protein metabolism in IS development. These specially modification proteins showing increased abundance may be involved in maintaining protein folding correction, removing non-functional protein, or refolding denatured proteins, during the grain-filling.

### Low Expression of Proteins Involved in Sugar to Starch Conversion in IS Leads to Developmental Stagnancy

**FIGURE 3:**
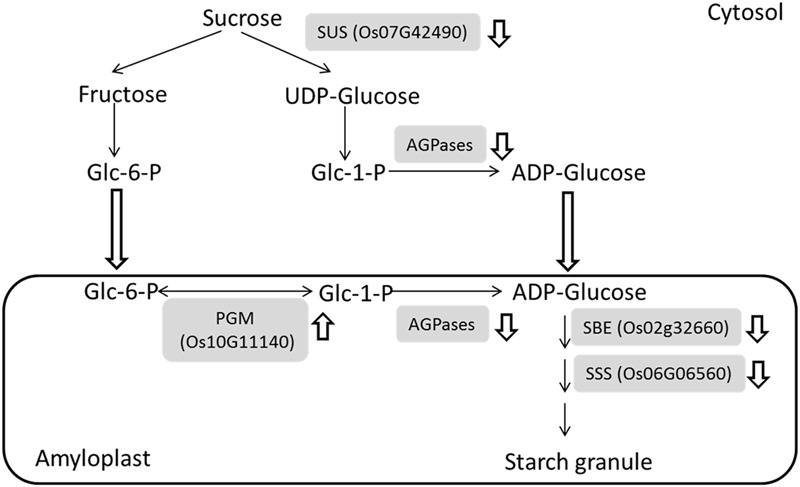
**Down-regulation of major metabolic processes in the sugar to starch conversion during the grain-filling of IS.** Four enzymes in the sugar to starch conversion pathways were identified with reduced abundances while an enzyme involved in starch synthesis was up-regulated. and indicates down-regulation and up-regulation, respectively. SuSase, Sucrose synthase; AGPase, ADP glucose pyrophosphorylase; SSS, Soluble starch synthase; SBE, Starch branching enzyme; PGM, phosphoglucomutase.

The greatest difference can be observed between IS and SS at 9 DAF due to the starch granule developmental stagnancy. Our comparative proteomic study revealed that sugar to starch conversion proteins including SuSase (Sucrose synthase, Os07g42490), AGPase (ADP glucose pyrophosphorylase, e.g., Os03g52460 and Os08g25734), SSS (Soluble starch synthase, Os06g06560), and SBE (Starch branching enzyme, Os02g32660) consistently exhibited decreased abundances in IS (**Supplementary Table S1**). The SuSase and AGPases are responsible for conversion of sucrose into ADP-glucose and are considered to be the most critical enzymes in starch synthesis (Tetlow, 2006; Zeeman et al., 2010). Subsequent catalysis by SSS and SBE contribute to the final formation of starch granule (Tetlow, 2006; Zhu et al., 2011), thereby the expression of the identified enzymes involved in sucrose to starch conversion were indicated and summarized in **Figure 3**. These observations were consistent with our previous work and other groups’ results. DNA microarray data showed that a group of starch synthesis-related genes had higher mRNA expressions in the SS than in IS during grain filling, which including the SUS genes *SUS2, SUS3*, and *SUS4*, the UGPase gene *UGP1*, the AGPase genes *AGPS1, AGPS2, AGPL2*, and *AGPL3*, the GBSS gene *GBSSI*, the SSS genes *SSI* and *SSIIa*, the SBE genes *BEI* and *BEIIb* (Zhu et al., 2011). Meanwhile, low activities of the starch synthesis enzymes, i.e., SUS, AGPase, SSS, and GBSS activities, were also observed in the inferior grains during grain filling in the rice and wheat plants (Jiang et al., 2003; Dai et al., 2009; Tang et al., 2009; Yang and Zhang, 2010). Thus, low mRNA levels, protein abundant and enzyme activities of starch synthesis pathway may be the key limiting factors for inferior grain filling, which reduced the conversion of sugars to starch, since the carbohydrate supply are comparable at the early beginning of grain filling in the IS and SS (Yang et al., 2006; Zhu et al., 2011). Interestingly, phosphoglucomutase (PGM, Os10g11140), an enzyme associated with starch synthesis, was up-regulated during the grain-filling in IS compared to SS. PGM catalyzes the reversible interconversions between glucose 6-phosphate (Glc-6-P) and glucose 1-phosphate (Glc-1-P) and provide Glc-1-P for AGPases reaction (Fritzius et al., 2001; Tetlow, 2006) (**Figure 3**). It probably maintains the Glc-6-P/Glc-1-P pool balance during their depletion of starch synthesis, further regulating the grain filling process between IS and SS.

### Energy Metabolism Is Essential for IS Development

**FIGURE 4:**
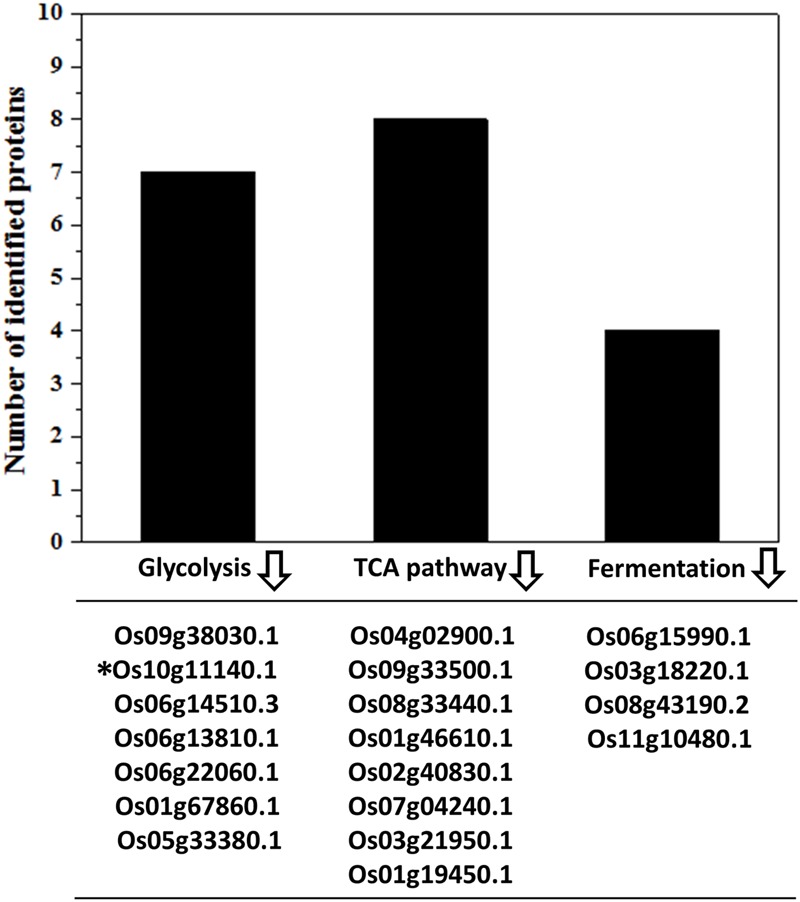
**Down-regulation of energy metabolism-related proteins in IS.** Functional classification and calculation of energy metabolism-related proteins in IS. Lower expression of proteins involved in the glycolysis, TCA cycles and fermentation pathways were listed and identified in the IS compared to SS. indicates down-regulation. ^∗^Represents only this protein of fold change > 1.2 with increased abundances.

The carbon and energy metabolism including the glycolysis and TCA cycle processes provide the energy and metabolites required for embryo establishment and seed development (Farré et al., 2001; Fernie et al., 2004; Carrari and Fernie, 2006). Glycolysis breaks down the glucose with the production of pyruvate and ATPs (Zhang et al., 2015). Subsequently, the glycolysis derived pyruvates should be taken in favor by TCA cycle to generate more ATPs (Zhang et al., 2015). The ATP contents in IS and SS were highly related to their grain filling rate (Yang et al., 1999). Slowed ATP supply in IS at the early grain-filling stage was detrimental to the sink establishment in the grains (Zhou et al., 1991). In this study, the relative expression of all identified proteins associated with glycolysis and TCA cycle were showed to be lower in the grain-filling process on IS than SS (**Figure 4**), which strongly indicate the low ATP contents in IS leading to the stagnant development in the grains. Generating more ATP and inter-metabolites during the glycolysis and TCA cycle is essential for the endosperm cell enlargement and starch synthesis (Xu et al., 2008; Zhang et al., 2014). Interestingly, abundance of four identified proteins involved in fermentation was also lower in IS compared to SS (**Figure 4**). For example, Os11g10480 plays an important role in the alcohol fermentation pathway and was considered to maintain an appropriate ATP level for starch synthesis during the low oxygen tension (Xu et al., 2008). Therefore, down-regulation of alcohol fermentation probably also contributes to lower ATP generation affecting normal starch synthesis in IS development.

### Increased Abundance of Seed-Storage Proteins Observed in the Inferior Spikelets

Rice seed-storage proteins are generally divided into albumins, prolamins, glutelins as well as globulins (Jiang et al., 2014). The major proteins present in most cereals are alcohol-soluble prolamins, whereas the alkaline-soluble glutelins constitute up to 60–80% of the total seed protein in rice grains (Yamamoto et al., 2006). In this study, two glutelin-type (e.g., Os02g15090 and Os02g16820), one globulin-type (Os03g46100) and two albumin superfamily (Os05g41970 and Os03g25350) seed storage proteins were identified in the differentially expressed proteins between IS and SS, among which consistently showed increasing abundance in the IS compared with the SS, indicating a dominant role of seed storage proteins in the IS. Interestingly, transcriptional level analysis between IS and SS also revealed the high expression of seed storage protein genes observed in the IS and suggested an inhibitory effects of seed storage proteins during the grain-filling process (Sekhar et al., 2015). Therefore, increased abundance of seed storage proteins in IS is probably preventing grain-filling rate through the retardation of developmental signaling cascades involved the grain-filling process. Genetic modification and improvement on genes encoding seed storage proteins would be helpful to improve the grain filling of IS. On the other hand, grain-filling in the SS reaches its peak whereas IS just begins the process of grain filling at 9 DAF, which may account for the high expression of seed storage proteins in IS compared to SS. However, the inhibitory effect or detailed regulation mechanism of seed storage proteins on grain filling process still needs further investigation.

### Correlation between Protein and Gene Expression and Comparison of Grain-Filling Proteomics Data in Plants

**FIGURE 5:**
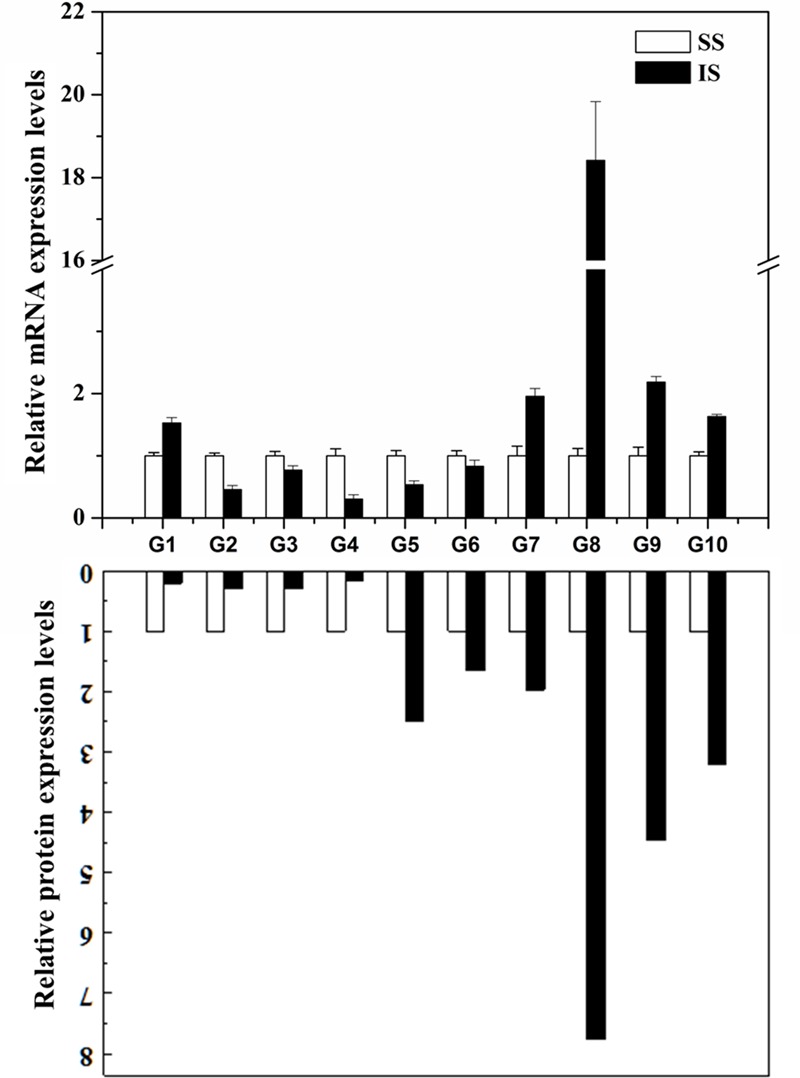
**Correlation between the protein and gene expression during the grain-filling in rice IS and SS.** Selected proteins identified by SWATH-MS were examined for changes at the transcript level by qRT-PCR analysis using the rice IS and SS at 9 DAF. Relative expression levels in IS were normalized against the SS. Bars represent SE (*n* = 3). The comparison of protein expression levels between IS and SS derived from proteomics data of this study. G1: Os07g49400; G2: Os11g26910; G3: Os01g46610; G4: Os01g67860; G5: Os02g21970; G6: Os06g51150; G7: Os05g15770; G8: Os03g25350; G9: Os04g53230; G10: Os10g32550.

To examine whether the differentially expressed proteins are associated with transcriptional changes, microarray or RNA-sequencing data from online database^2^ and the literature (Zhu et al., 2011; Sekhar et al., 2015; Sun et al., 2015) were initially queried. However, the microarray or RNA-sequencing gene expression data for the grain-filling of IS and SS do not contain many corresponding proteins identified from this SWATH-MS study, indicating a low congruency of proteomic and transcriptional profiles as reported from other proteomics investigations (Gallardo et al., 2007; Lan et al., 2011; Zhang et al., 2012). Here, the expression levels of 10 selected genes involved in variety of processes were analyzed by qRT-PCR. As shown in **Figure 5**, eight of the selected genes showed up- or down-regulated expression during the grain-filling between IS and SS, consistent with the changes in abundances of the corresponding proteins as revealed from the SWATH-MS experiment (**Supplementary Table S1**). On the other hand, the expression levels of Os07g49400 and Os06g51150 were found to have upregulated and no significant changes in the IS compared to SS, respectively. However, reduced abundance of Os07g49400 and increased abundances of Os06g51150 were detected in the proteomes between IS and SS in this study. Thus, the differential expression levels of their corresponding proteins are likely to be resulting from varying post-transcriptional regulation such as alternative splicing (AS), RNA transport or translation efficiency (Lan et al., 2011). Meanwhile, we also examined the differential expressed proteins showed in this study with its corresponding gene expression analysis on grain-filing process between IS and SS by other groups (Sekhar et al., 2015; Sun et al., 2015). Approximately 42% of proteome changes were consistent with gene expression changes whereas 10% of their corresponding gene expressions showed no significant changes and the rest of them exhibited contrast changes (**Supplementary Table S3**), further indicating that the grain filling process between IS and SS was probably regulated by post-transcriptional modification (PTM).

## Discussion

### Few Transcription Factors Are Detected in Quantitative Proteomics

Large scale expression profiling analysis indicates that transcriptome reprogramming is pervasive during rice grain filling. Therefore, transcription factors (TFs) have become research hotspots during grain filling process in the past decade (Zhu et al., 2003). TFs of multiple families have been demonstrated to control yield potential of cereal plants (Zuo and Li, 2013). For example, basic leucine zipper factor, RISBZ1 and prolamin box binding factor (RPBF) were observed to coordinately control seed storage protein accumulation, subsequently to affect grain starch content in rice grain (Kawakatsu et al., 2009). The overexpression of APETELA2 (AP2) domain containing protein, AP37, was reported to increase yield under drought condition (Oh et al., 2009). Furthermore, a SBP-domain TF (OsSPL16) was recently found to regulate the expression of GW7 allele and also improved the grain yield and quality (Wang et al., 2015). However, few TFs were reported to control the differential grain filling percentages between superior and IS. From our dataset, only one NAC domain-containing TF (Os05g34310) was detected, indicating its potential role in regulating rice grain filling of two types of spikelets. Although several NAC TFs have been documented to improve protein and mineral content in cereal grains (Uauy et al., 2006; Sperotto et al., 2009), their molecular function remains unclear. Further molecular and genetic studies are required to unravel the underlying mechanisms of this TF during rice grain filling. In addition, the presence of few TFs in proteomic data indicates that the regulations of these genes are not limited at the protein level. Post-transcriptional (e.g., AS) and post-translational (phosphorylation and glycosylation) modifications may also regulate the isoforms or activities of TFs during rice grain filling.

### RNA-Processing Contributes to Differential Grain Filling Potential in SS and IS

Before export into cytoplasm, messenger RNA is processed by a complex of proteins and undergoes 5′-capping, splicing and 3′-tailing. Amongst them, AS has drawn increasingly attention recently. AS can generate multiple mRNA isoforms from a single pre-mRNA (Reddy et al., 2013). Studies in animals revealed that this type of regulation greatly increase the transcriptome and subsequent proteome diversity. It has been estimated that over 95% of intron-containing genes are alternative spliced (Reddy et al., 2013). The biological significance of AS has two major outcomes. The first one is that AS can regulate expression level of genes by producing unstable mRNA isoforms. A large portion of mRNA isoforms will be degraded by non-sense-mediated decay or microRNAs (Drechsel et al., 2013). The rest of them will be translated into protein isoforms with altered subcellular localization, stability, or functionality. New findings have suggested that certain genes undergo AS to show differential regulation in stress response, organ development and circadian rhythm (Staiger and Brown, 2013), but its role in rice grain filling is unclear. By using next-generation of deep sequencing, recent studies suggested that approximately 60% intron-containing genes undergo AS in plants such as *Arabidopsis*, soybean, and maize (Marquez et al., 2012; Shen et al., 2014; Thatcher et al., 2014), indicating a large and unchartered regulatory networks in plant remain to be elucidated. Interestingly, 12 differentially expressed proteins were found from our proteomic dataset (**Supplementary Table S1**), suggesting RNA processing is an important step to regulate grain filling between IS and SS. Definitely, further study is required to dissect the role of these proteins during rice grain filling.

### Combinatory Approaches Are Needed to Resolve the Complex Nature of Rice Grain Filling Process

Given that the complexity of the grain filling process using a single approach it is hard to fully understand the underlying mechanisms. Using of combinatory approach such as proteogenomics, an analytical scheme that combines transcriptome and proteomic analysis, is more reliable to study the regulatory mechanisms at both transcripts and protein levels with a better self-annotation (Komor et al., 2016). In addition, single molecule long-read sequencing has become a powerful method to get full-length transcripts for gene model re-annotation and subsequent AS analysis. Coupling with other techniques such as ribosome sequencing (Brar and Weissman, 2015), positional proteomics (Marino et al., 2015), and peptidome (Tavormina et al., 2015) may further increase our understanding on genome coding ability and its regulation during rice grain filling.

## Conclusion

Proteomics investigations have provided comprehensive evaluations of proteome changes in plants responding to different biological processes and environmental stresses. However, proteome information on grain-filling is still fragmented and most studies have focused on relatively small amounts of proteome changes using conventional gel-based proteomics approaches (Zhang et al., 2015; Chen et al., 2016). The current study utilizes the SWATH-MS platform to accurately quantify changes in protein abundance during the grain-filling between IS and SS. Analysis of changes in the global protein profile revealed several components of biological processes in IS development. Slow-down of cellular processes including photosynthetic rate, energy and protein metabolism as well as the sugar to starch conversion were observed in the IS compared to the SS, which would undoubtedly result in poor grain filling in the IS. Increased abundance of seed storage proteins and several RNA-processing proteins may also shed light on the reason for developmental stagnancy in the IS. Therefore, our SWATH-MS based proteomic study in combination with further functional investigations would provide novel insights into the control mechanism of rice grain filling and find out crucial regulators for potential applications to increase rice yield in future.

## Author Contributions

F-YZ, M-XC, GZ, and JZ designed research scheme, F-YZ, M-XC, Y-WS, XX, N-HY, Y-YC, T-YL, H-XL, G-QW, W-LC, YJ, and Y-HG performed experiments, F-YZ, M-XC, and N-HY analyzed data, F-YZ and M-XC wrote the manuscript, F-YZ, M-XC, Y-WS, XX, SL, XP, LC, GZ, and JZ revised it. All authors read and approved the final manuscript.

## Conflict of Interest Statement

The authors declare that the research was conducted in the absence of any commercial or financial relationships that could be construed as a potential conflict of interest.

## Footnotes

1 http://ppdb.tc.cornell.edu/dbsearch/mapman.aspx

2 https://www.genevestigator.com/gv/

## Supplementary Material

TABLE S1 **List of differentially expressed proteins between superior and inferior spikelets in grain-filling stage**.

TABLE S2 **Peptide and protein reports for the proteins quantified by SWATH-MS**.

TABLE S3 **Correlation between the proteomic and RNA-seq data**.
