# Supplementary material for: SWATH-MS Quantitative Analysis of Proteins in the Rice Inferior and Superior Spikelets during Grain Filling
Source: Front Plant Sci. 2016 Dec 20;7:1926. doi: 10.3389/fpls.2016.01926 (PMC5169098; doi:10.3389/fpls.2016.01926)
Supplement: TABLE S1 — List of differentially expressed proteins between superior and inferior spikelets in grain-filling stage. [file Table_1.DOCX]

**Table 1. List of differentially expressed proteins between superior and inferior spikelets in grain-filling stage**

**p-Val**

**Fold change**

**Functional classification**

**Annotation**

**Accession ID**

**Uniprot**

| **Amino acid metabolism** | | | |  |  |  |
| --- | --- | --- | --- | --- | --- | --- |
| B8B8K7 | Os07g42600.1 | alanine aminotransferase 2, putative, expressed | | 13.1.1.3.1 amino acid metabolism.synthesis.central amino acid metabolism.alanine.alanine aminotransferase | 0.754 | 0.011 |
| B8BGM4 | Os10g25130.1 | alanine aminotransferase 2, putative, expressed | | 13.1.1.3.1 amino acid metabolism.synthesis.central amino acid metabolism.alanine.alanine aminotransferase | 1.212 | 0.000 |
| A2WP83 | Os01g22010.4 | S-adenosylmethionine synthetase, putative, expressed | | 13.1.2.3 amino acid metabolism.synthesis | 1.247 | 0.025 |
| B8AH93 | Os02g47590.1 | ornithine carbamoyltransferase, chloroplast precursor, putative, expressed | | 13.1.2.3.21 amino acid metabolism.synthesis.glutamate family.arginine.ornithine carbamoyltransferase | 0.611 | 0.037 |
| B8AM24 | Os03g18810.1 | transaminase/ transferase, transferring nitrogenous groups, putative, expressed | | 13.1.3.5.3 amino acid metabolism.synthesis.aspartate family.lysine.LL-diaminopimelic acid aminotransferase; 13.1.6.2 amino acid metabolism.synthesis.aromatic aa.phenylalanine and tyrosine | 0.721 | 0.004 |
| B8AK88 | Os03g55280.1 | aspartate-semialdehyde dehydrogenase, putative, expressed | | 13.1.3.6.1.2 amino acid metabolism.synthesis.aspartate family.misc.homoserine.aspartate semialdehyde dehydrogenase | 1.444 | 0.036 |
| B8AWV4 | Os05g49800.1 | ketol-acid reductoisomerase, chloroplast precursor, putative, expressed | | 13.1.4.1 amino acid metabolism.synthesis.branched chain group.common | 0.764 | 0.001 |
| B8AFH3 | Os02g39570.4 | acetolactate synthase/ amino acid binding protein, putative, expressed | | 13.1.4.1.1 amino acid metabolism.synthesis.branched chain group.common.acetolactate synthase | 0.239 | 0.015 |
| B8BJX9 | Os11g14950.1 | acetolactate synthase/ amino acid binding protein, putative, expressed | | 13.1.4.1.1 amino acid metabolism.synthesis.branched chain group.common.acetolactate synthase | 0.517 | 0.000 |
| A2XYC2 | Os04g55720.1 | D-3-phosphoglycerate dehydrogenase, chloroplast precursor, putative, expressed | | 13.1.5.1.1 amino acid metabolism.synthesis.serine-glycine-cysteine group.serine.phosphoglycerate dehydrogenase | 0.784 | 0.000 |
| A2ZDY4 | Os11g26850.2 | adenosylhomocysteinase, putative, expressed | | 13.2.3.4 amino acid metabolism.degradation.aspartate family.methionine | 0.536 | 0.000 |
| B8AUI9 | Os04g53230.1 | aminomethyltransferase, mitochondrial precursor, putative, expressed | | 13.2.5.2 amino acid metabolism.degradation.serine-glycine-cysteine group.glycine | 4.458 | 0.001 |
| **Cell** |  |  | |  |  |  |
| A2XLK9 | Os03g51600.1 | tubulin alpha-3 chain, putative, expressed | | 31.1 cell.organisation | 0.717 | 0.001 |
| A2XNF8 | Os04g46910.1 | actin-depolymerizing factor, putative, expressed | | 31.1 cell.organisation | 0.712 | 0.000 |
| A2YG29 | Os06g46000.1 | tubulin beta-3 chain, putative, expressed | | 31.1 cell.organisation | 0.706 | 0.000 |
| A2Z4X9 | Os09g33810.2 | ankyrin repeat domain-containing protein 2, putative, expressed | | 31.1 cell.organisation | 1.409 | 0.003 |
| A2YGV2 | Os06g49480.2 | peptidyl-prolyl cis-trans isomerase CYP19-4 precursor, putative, expressed | | 31.3.1 cell.cycle.peptidylprolyl isomerase | 1.297 | 0.013 |
| A2ZH90 | Os11g01380.1 | clathrin heavy chain, putative, expressed | | 31.4 cell.vesicle transport | 0.609 | 0.000 |
| B8BJD1 | Os11g07280.1 | coatomer subunit beta, putative, expressed | | 31.4 cell.vesicle transport | 0.711 | 0.000 |
| **Cell wall** | |  | |  |  |  |
| B8BPH4 | Os12g25690.1 | UDP-glucose 6-dehydrogenase, putative, expressed | | 10.1.4 cell wall.precursor synthesis.UGD; 34.18 transport.unspecified anions | 0.560 | 0.000 |
| A2XWM8 | Os04g48490.1 | fasciclin-like arabinogalactan protein 10 precursor, putative, expressed | | 10.5.1 cell wall.cell wall proteins.AGPs | 0.731 | 0.000 |
| A6N1F0 | Os03g40270.1 | alpha-1,4-glucan-protein synthase, putative, expressed | | 10.5.5 cell wall.cell wall proteins.RGP | 0.181 | 0.000 |
| A2XM08 | Os03g53800.5 | periplasmic beta-glucosidase precursor, putative, expressed | | 10.6.1 cell wall.degradation.cellulases and beta -1,4-glucanases | 0.740 | 0.006 |
| B8AIS2 | Os02g51620.1 | beta-D-xylosidase, putative, expressed | | 10.6.2 cell wall.degradation.mannan-xylose-arabinose-fucose | 0.262 | 0.002 |
| **Development** | |  | |  |  |  |
| A2WXY9 | Os01g67310.1 | patatin class 1 precursor, putative, expressed | | 33.1 development.storage proteins | 1.257 | 0.038 |
| A2X2Z1 | Os02g15090.1 | glutelin type-B 7 precursor, putative, expressed | | 33.1 development.storage proteins | 1.216 | 0.000 |
| B8AEZ3 | Os02g16820.1 | glutelin type-B 4 precursor, putative, expressed | | 33.1 development.storage proteins | 1.265 | 0.000 |
| A2X399 | Os02g16830.1 | glutelin type-B 4 precursor, putative, expressed | | 33.1 development.storage proteins | 1.602 | 0.000 |
| A2XGF3 | Os03g21790.1 | cupin family protein, expressed | | 33.1 development.storage proteins | 1.520 | 0.013 |
| T1T5D8 | Os03g31360.1 | glutelin type-A 3 precursor, putative, expressed | | 33.1 development.storage proteins | 1.242 | 0.000 |
| A2XKC1 | Os03g46100.1 | globulin-1 S allele precursor, putative, expressed | | 33.1 development.storage proteins | 1.607 | 0.000 |
| A2YQV0 | Os08g03410.1 | glutelin, putative, expressed | | 33.1 development.storage proteins | 1.279 | 0.002 |
| B8AN79 | Os03g62620.4 | late embryogenesis abundant protein, putative, expressed | | 33.2 development.late embryogenesis abundant | 0.707 | 0.003 |
| B8AAS8 | Os01g39310.1 | protein SEY1, putative, expressed | | 33.99 development.unspecified | 0.698 | 0.000 |
| A2XE22 | Os03g12230.2 | caleosin, putative, expressed | | 33.99 development.unspecified | 1.796 | 0.018 |
| A2Y939 | Os06g04990.1 | early nodulin 93, putative, expressed | | 33.99 development.unspecified | 1.515 | 0.026 |
| A2YD98 | Os06g30370.1 | OsMFT1 - Rice MFT-Like1 homogous to Arabidopsis Mother of FT and TFL1 gene, expressed | | 33.99 development.unspecified | 2.311 | 0.000 |
|  |  |  | |  |  |  |
| **DNA** |  |  | |  |  |  |
| B8AXA2 | Os05g02300.1 | histone H2A, putative, expressed | | 28.1.3 DNA.synthesis/chromatin structure.histone | 0.510 | 0.001 |
| A2WWR4 | Os05g38740.1 | histone H4, putative, expressed | | 28.1.3 DNA.synthesis/chromatin structure.histone | 0.696 | 0.000 |
| B8A9Q0 | Os01g36090.1 | DNA-damage-repair/toleration protein DRT102, putative, expressed | | 28.2 DNA.repair | 0.447 | 0.007 |
| **Fermentation** | |  | |  |  |  |
| A2YBK1 | Os06g15990.1 | aldehyde dehydrogenase, mitochondrial precursor, putative, expressed | | 5.10 fermentation.aldehyde dehydrogenase | 0.565 | 0.005 |
| B8AM29 | Os03g18220.1 | pyruvate decarboxylase isozyme 2, putative, expressed | | 5.2 fermentation.PDC | 0.468 | 0.000 |
| B8B9C5 | Os08g43190.2 | sorbitol dehydrogenase, putative, expressed | | 5.3 fermentation.ADH | 0.713 | 0.000 |
| D7PPJ0 | Os11g10480.1 | alcohol dehydrogenase 1, putative, expressed | | 5.3 fermentation.ADH | 0.752 | 0.020 |
| **Gluconeogenese** | |  | |  |  |  |
| B8AEK0 | Os02g13840.1 | citrate synthase, glyoxysomal precursor, putative, expressed | | 6.1 gluconeogenese/ glyoxylate cycle.citrate synthase | 0.475 | 0.027 |
| A2XSR2 | Os04g31700.1 | methylisocitrate lyase 2, putative, expressed | | 6.9 gluconeogenese/ glyoxylate cycle.isocitrate lyase | 0.657 | 0.014 |
| Glycolysis | |  | |  |  |  |
| A4KC21 | Os09g38030.1 | UTP--glucose-1-phosphate uridylyltransferase, putative, expressed | | 4.1 glycolysis.UGPase | 0.732 | 0.000 |
| B8BG13 | Os10g11140.1 | phosphoglucomutase, chloroplast precursor, putative, expressed | | 4.2 glycolysis.PGM | 1.557 | 0.013 |
| B8B0B0 | Os06g14510.3 | glucose-6-phosphate isomerase, cytosolic B, putative, expressed | | 4.3 glycolysis.G6PIsomerase | 0.777 | 0.003 |
| A2YB91 | Os06g13810.1 | pyrophosphate--fructose 6-phosphate 1-phosphotransferase beta subunit, putative, expressed | | 4.5 glycolysis.pyrophosphate-fructose-6-P phosphotransferase | 0.749 | 0.001 |
| B8B1A6 | Os06g22060.1 | pyrophosphate--fructose 6-phosphate 1-phosphotransferase alpha subunit, putative, expressed | | 4.5 glycolysis.pyrophosphate-fructose-6-P phosphotransferase | 0.718 | 0.003 |
| B8A7T3 | Os01g67860.1 | fructose-bisphosphate aldolase cytoplasmic isozyme, putative, expressed | | 4.7 glycolysis.aldolase | 0.151 | 0.000 |
| B8AY35 | Os05g33380.1 | fructose-bisphosphate aldolase cytoplasmic isozyme, putative, expressed | | 4.7 glycolysis.aldolase | 0.721 | 0.001 |
| **Hormone metabolism** | |  | |  |  |  |
| A2Y3H0 | Os05g28210.1 | embryonic abundant protein 1, putative, expressed | | 17.1.3 hormone metabolism.abscisic acid.induced-regulated-responsive-activated | 1.295 | 0.033 |
| A2XC88 | Os03g04340.1 | 24-methylenesterol C-methyltransferase 2, putative, expressed | | 17.3.1.2.2 hormone metabolism.brassinosteroid.synthesis-degradation.sterols.SMT | 0.349 | 0.000 |
| A2Z6Y9 | Os10g25780.3 | cell elongation protein diminuto, putative, expressed | | 17.3.1.2.8 hormone metabolism.brassinosteroid.synthesis-degradation.sterols.DWF1 | 0.723 | 0.023 |
| A2YH94 | Os07g01020.1 | pyridoxin biosynthesis protein ER1, putative, expressed | | 17.5.3 hormone metabolism.ethylene.induced-regulated-responsive-activated; 18 Co-factor and vitamine metabolism | 0.624 | 0.007 |
| **Lipid metabolism** | |  | |  |  |  |
| A2Y2U1 | Os05g22940.1 | acetyl-coenzyme A carboxylase, putative, expressed | | 11.1.1 lipid metabolism.FA synthesis and FA elongation.Acetyl CoA Carboxylation | 0.630 | 0.045 |
| B8BIC8 | Os10g42100.1 | pyruvate kinase isozyme G, chloroplast precursor, putative, expressed | | 11.1.30 lipid metabolism.FA synthesis and FA elongation.pyruvate kinase | 0.536 | 0.000 |
| A2YSI3 | Os08g12840.1 | 3-hydroxyacyl-[acyl-carrier protein] dehydratase/ catalytic, putative, expressed | | 11.1.5 lipid metabolism.FA synthesis and FA elongation.beta hydroxyacyl ACP dehydratase | 0.372 | 0.000 |
| A2ZHF7 | Os12g02340.1 | LTPL14 - Protease inhibitor/seed storage/LTP family protein precursor, expressed | | 11.6 lipid metabolism.lipid transfer proteins etc | 0.543 | 0.000 |
| A6MZG5 | Os11g39220.2 | acyl-coenzyme A oxidase 1, putative, expressed | | 11.9.4.2 lipid metabolism.lipid degradation.beta-oxidation.acyl CoA DH | 1.660 | 0.028 |
| B8AFN7 | Os02g17390.1 | peroxisomal fatty acid beta-oxidation multifunctional protein, putative, expressed | | 11.9.4.9 lipid metabolism.lipid degradation.beta-oxidation.mutifunctional | 0.505 | 0.015 |
| **Major CHO metabolism** | |  | |  |  |  |
| P93430 | Os01g44220.6 | glucose-1-phosphate adenylyltransferase large subunit 1, chloroplast precursor, putative, expressed | | 2.1.2.1 major CHO metabolism.synthesis.starch.AGPase | 0.793 | 0.000 |
| B8AR31 | Os03g52460.1 | glucose-1-phosphate adenylyltransferase large subunit 3, chloroplast precursor, putative, expressed | | 2.1.2.1 major CHO metabolism.synthesis.starch.AGPase | 0.646 | 0.003 |
| A2YU91 | Os08g25734.1 | glucose-1-phosphate adenylyltransferase small subunit, chloroplast precursor, putative, expressed | | 2.1.2.1 major CHO metabolism.synthesis.starch.AGPase | 0.701 | 0.000 |
| D0TZM8 | Os06g06560.1 | soluble starch synthase 1, chloroplast precursor, putative, expressed | | 2.1.2.2 major CHO metabolism.synthesis.starch.starch synthase | 0.739 | 0.001 |
| D0TZK1 | Os02g32660.1 | 1,4-alpha-glucan branching enzyme IIB, chloroplast precursor, putative, expressed | | 2.1.2.3 major CHO metabolism.synthesis.starch.starch branching | 0.676 | 0.000 |
| D0TZF9 | Os08g40930.1 | isoamylase, putative, expressed | | 2.1.2.4 major CHO metabolism.synthesis.starch.debranching | 0.610 | 0.001 |
| B8AHM8 | Os02g48720.2 | ADP,ATP carrier protein, mitochondrial precursor, putative, expressed | | 2.1.2.5 major CHO metabolism.synthesis.starch.transporter | 0.607 | 0.000 |
| A2Y2Y3 | Os05g23720.1 | ADP,ATP carrier protein, mitochondrial precursor, putative, expressed | | 2.1.2.5 major CHO metabolism.synthesis.starch.transporter | 0.274 | 0.007 |
| A2YNQ2 | Os07g42490.2 | sucrose synthase 3, putative, expressed | | 2.2.1.5 major CHO metabolism.degradation.sucrose.Susy | 0.749 | 0.000 |
| **Minor CHO metabolism** | |  | |  |  |  |
| B8BHM9 | Os10g35110.2 | alpha-galactosidase precursor, putative, expressed | | 3.8.2 minor CHO metabolism.galactose.alpha-galactosidases | 0.727 | 0.001 |
| **Mitochondrial electron transport** | | | | |  |  |
| B8AEU4 | Os02g57180.1 | NADH-ubiquinone oxidoreductase 39 kDa subunit, mitochondrial precursor, putative, expressed | 9.1.2 mitochondrial electron transport / ATP synthesis.NADH-DH.localisation not clear | | 0.782 | 0.000 |
| B8BG64 | Os10g17280.1 | ATP synthase gamma chain, mitochondrial precursor, putative, expressed | 9.9 mitochondrial electron transport / ATP synthesis.F1-ATPase | | 0.545 | 0.017 |
| **MISC** |  |  |  | |  |  |
| B8B6R2 | Os07g02200.1 | blue copper protein precursor, putative, expressed | 26.19 misc.plastocyanin-like | | 0.560 | 0.007 |
| A2WW14 | Os01g58660.1 | LTPL29 - Protease inhibitor/seed storage/LTP family protein precursor, expressed | 26.21 misc.protease inhibitor/seed storage/lipid transfer protein (LTP) family protein | | 0.422 | 0.000 |
| A2XH60 | Os03g25350.1 | 5a2 protein, putative, expressed | 26.21 misc.protease inhibitor/seed storage/lipid transfer protein (LTP) family protein | | 7.753 | 0.000 |
| B8BKT7 | Os11g32260.1 | lysosomal alpha-mannosidase precursor, putative, expressed | 26.3 misc.gluco-, galacto- and mannosidases | | 0.740 | 0.004 |
| **Nucleotide metabolism** | | |  | |  |  |
| B8AMR2 | Os03g61600.1 | phosphoribosylformylglycinamidine cyclo-ligase,chloroplast/mitochondrial precursor, putative, expressed | 23.1.2.5 nucleotide metabolism.synthesis.purine.AIR synthase | | 0.501 | 0.009 |
| B8AI32 | Os02g50350.1 | dihydropyrimidine dehydrogenase precursor, putative, expressed | 23.2 nucleotide metabolism.degradation | | 0.741 | 0.010 |
| A2X7E9 | Os02g41590.1 | adenosine kinase 2, putative, expressed | 23.3.2.1 nucleotide metabolism.salvage.nucleoside kinases.adenosine kinase | | 1.257 | 0.002 |
| A2ZLE4 | Os12g36194.1 | nucleoside diphosphate kinase 2, chloroplast precursor, putative, expressed | 23.4.10 nucleotide metabolism.phosphotransfer and pyrophosphatases.nucleoside diphosphate kinase | | 0.452 | 0.001 |
| B8ARU1 | Os04g59040.1 | soluble inorganic pyrophosphatase, putative, expressed | 23.4.99 nucleotide metabolism.phosphotransfer and pyrophosphatases.misc | | 0.515 | 0.000 |
| **Not assigned** | |  |  | |  |  |
| A2WKG3 | Os01g04650.1 | PB1 domain containing protein, expressed | 35.1 not assigned.no ontology | | 2.377 | 0.002 |
| A2X7K2 | Os02g42290.3 | OsClp3 - Putative Clp protease homologue, expressed | 35.1 not assigned.no ontology | | 0.730 | 0.000 |
| B8AJS5 | Os03g29240.1 | PDI-like protein, putative, expressed | 35.1 not assigned.no ontology | | 0.727 | 0.001 |
| A2XMQ2 | Os03g57420.1 | ML domain protein, putative, expressed | 35.1 not assigned.no ontology | | 0.641 | 0.005 |
| A2XXP3 | Os04g52960.1 | nucleolin, putative, expressed | 35.1 not assigned.no ontology | | 0.786 | 0.000 |
| A6MZS5 | Os06g02220.1 | MTA/SAH nucleosidase, putative, expressed | 35.1 not assigned.no ontology | | 0.657 | 0.010 |
| A2YAN2 | Os06g11090.1 | gibberellin receptor GID1L2, putative, expressed | 35.1 not assigned.no ontology | | 1.362 | 0.001 |
| B8B5L9 | Os07g46370.1 | EMB2221, putative, expressed | 35.1 not assigned.no ontology | | 0.679 | 0.049 |
| A2YPT8 | Os07g47510.1 | stress-related protein, putative, expressed | 35.1 not assigned.no ontology | | 0.571 | 0.000 |
| B8BIM7 | Os11g01600.2 | macrophage migration inhibitory factor, putative, expressed | 35.1 not assigned.no ontology | | 3.167 | 0.000 |
| B8AD25 | Os01g03760.1 | expressed protein | 35.2 not assigned.unknown | | 1.410 | 0.001 |
| A2XAL2 | Os02g55440.2 | transmembrane 9 superfamily protein member 2 precursor, putative, expressed | 35.2 not assigned.unknown | | 0.218 | 0.028 |
| A2XT28 | Os04g33150.1 | desiccation-related protein PCC13-62 precursor, putative, expressed | 35.2 not assigned.unknown | | 1.385 | 0.000 |
| A2XTC1 | Os04g34100.1 | expressed protein | 35.2 not assigned.unknown | | 0.754 | 0.029 |
| A2Y665 | Os05g41970.1 | SSA1 - 2S albumin seed storage family protein precursor, expressed | 35.2 not assigned.unknown | | 1.262 | 0.000 |
| B8AW66 | Os05g46300.1 | F-box domain containing protein | 35.2 not assigned.unknown | | 2.337 | 0.029 |
| Q9ZNS9 | Os05g49440.2 | DUF1264 domain containing protein, putative, expressed | 35.2 not assigned.unknown | | 2.696 | 0.015 |
| A2YEZ8 | Os06g41030.1 | secreted protein, putative, expressed | 35.2 not assigned.unknown | | 0.371 | 0.001 |
| B8B8F6 | Os07g11630.1 | alpha-amylase/trypsin inhibitor precursor, putative, expressed | 35.2 not assigned.unknown | | 2.786 | 0.000 |
| A2YJG7 | Os07g11650.1 | alpha-amylase/trypsin inhibitor precursor, putative, expressed | 35.2 not assigned.unknown | | 1.363 | 0.000 |
| A2Z553 | Os10g06690.1 | expressed protein | 35.2 not assigned.unknown | | 0.527 | 0.027 |
| B8BGS7 | Os10g27180.1 | expressed protein | 35.2 not assigned.unknown | | 0.780 | 0.000 |
| B8BHC3 | Os10g32680.1 | expressed protein | 35.2 not assigned.unknown | | 1.617 | 0.001 |
| A2Z898 | Os10g32720.1 | plasma membrane associated protein, putative, expressed | 35.2 not assigned.unknown | | 0.337 | 0.000 |
| A2XF04 | Os12g10320.1 | plant-specific domain TIGR01627 family protein, expressed | 35.2 not assigned.unknown | | 0.584 | 0.004 |
| B8BMG2 | Os12g36880.1 | major pollen allergen Bet v 1-D/H, putative, expressed | 35.2 not assigned.unknown | | 0.534 | 0.000 |
| **Photosynthesis** | |  |  | |  |  |
| A6N154 | Os03g39610.1 | chlorophyll a-b binding protein, chloroplast precursor, putative, expressed | 1.1.1.1 PS.lightreaction.photosystem II.LHC-II | | 0.658 | 0.000 |
| A2XU61 | Os04g38410.1 | chlorophyll a-b binding protein CP24, chloroplast precursor, putative, expressed | 1.1.1.1 PS.lightreaction.photosystem II.LHC-II | | 0.712 | 0.000 |
| A2YML1 | Os07g37240.1 | chlorophyll A-B binding protein, putative, expressed | 1.1.1.1 PS.lightreaction.photosystem II.LHC-II | | 0.719 | 0.000 |
| A2YVI8 | Os08g33820.1 | chlorophyll a-b binding protein 4, chloroplast precursor, putative, expressed | 1.1.1.1 PS.lightreaction.photosystem II.LHC-II | | 0.334 | 0.003 |
| A2ZD01 | Os11g13890.1 | chlorophyll a-b binding protein M9, chloroplast precursor, putative, expressed | 1.1.1.1 PS.lightreaction.photosystem II.LHC-II | | 0.749 | 0.000 |
| B8A8L8 | Os01g31690.1 | oxygen-evolving enhancer protein 1, chloroplast precursor, putative, expressed | 1.1.1.2 PS.lightreaction.photosystem II.PSII polypeptide subunits | | 1.400 | 0.001 |
| J7EYM0 | Os10g41689.1 | photosystem II D2 protein, putative | 1.1.1.2 PS.lightreaction.photosystem II.PSII polypeptide subunits | | 0.348 | 0.000 |
| J3RPW4 | Os02g24632.1 | photosystem II 44 kDa reaction center protein, putative, expressed | 1.1.2.2 PS.lightreaction.photosystem | | 0.482 | 0.000 |
| J3RG68 | Os06g46436.1 | | 1.1.2.2 PS.lightreaction.photosystem I | | 0.527 | 0.001 |
| A2YKQ6 | Os07g25430.1 | photosystem I reaction center subunit IV A, chloroplast precursor, putative, expressed | 1.1.2.2 PS.lightreaction.photosystem I.PSI polypeptide subunits | | 0.794 | 0.000 |
| A2YY54 | Os08g44680.1 | photosystem I reaction center subunit II, chloroplast precursor, putative, expressed | 1.1.2.2 PS.lightreaction.photosystem I.PSI polypeptide subunits | | 0.492 | 0.000 |
| Q0PMC4 | Os10g21192.1 | photosystem Q, putative, expressed | 1.1.4 PS.lightreaction. | | 0.691 | 0.000 |
| B8A7M8 | Os01g49190.1 | ATP synthase beta chain, mitochondrial precursor, putative, expressed | 1.1.4 PS.lightreaction.ATP synthase | | 0.718 | 0.000 |
| J3R7S9 | Os04g16740.1 | ATP synthase alpha chain, putative, expressed | 1.1.4 PS.lightreaction.ATP synthase | | 0.468 | 0.000 |
| A2YLT1 | Os07g32880.1 | ATP synthase gamma chain, chloroplast precursor, putative, expressed | 1.1.4 PS.lightreaction.ATP synthase | | 0.480 | 0.000 |
| D3DF43 | Os09g08910.1 | ATP synthase alpha chain, mitochondrial, putative, expressed | 1.1.4 PS.lightreaction.ATP synthase | | 0.603 | 0.000 |
| B8B107 | Os06g45120.1 | vacuolar ATP synthase catalytic subunit A, putative, expressed | 1.1.4 PS.lightreaction.ATP synthase; 34.1.1 transport.p- and v-ATPases.H+-transporting two-sector ATPase | | 0.727 | 0.006 |
| B8AKX5 | Os03g57220.2 | hydroxyacid oxidase 1, putative, expressed | 1.2.2 PS.photorespiration.glycolate oxydase | | 0.572 | 0.002 |
| A2YCP9 | Os12g22030.1 | serine hydroxymethyltransferase, mitochondrial precursor, putative, expressed | 1.2.5 PS.photorespiration.serine hydroxymethyltransferase | | 0.553 | 0.015 |
| A2ZJQ0 | Os12g19470.2 | ribulose bisphosphate carboxylase small chain C, chloroplast precursor, putative, expressed | 1.3.2 PS.calvin cyle.rubisco small subunit | | 0.571 | 0.000 |
| A2Y650 | Os05g41640.2 | phosphoglycerate kinase, chloroplast precursor, putative, expressed | 1.3.3 PS.calvin cyle.phosphoglycerate kinase | | 0.717 | 0.000 |
| A2XC18 | Os03g03720.1 | glyceraldehyde-3-phosphate dehydrogenase B, chloroplast precursor, putative, expressed | 1.3.4 PS.calvin cyle.GAP; 4.9 glycolysis.glyceraldehyde 3-phosphate dehydrogenase | | 0.727 | 0.002 |
| A2XU83 | Os04g38600.3 | glyceraldehyde-3-phosphate dehydrogenase A, chloroplast precursor, putative, expressed | 1.3.4 PS.calvin cyle.GAP; 4.9 glycolysis.glyceraldehyde 3-phosphate dehydrogenase | | 0.780 | 0.044 |
| B8ACY2 | Os01g02880.1 | fructose-bisphosphate aldolase, chloroplast precursor, putative, expressed | 1.3.6 PS.calvin cyle.aldolase | | 0.436 | 0.000 |
| A2ZBX1 | Os11g07020.1 | fructose-bisphosphate aldolase, chloroplast precursor, putative, expressed | 1.3.6 PS.calvin cyle.aldolase | | 0.575 | 0.000 |
| A2Y8X8 | Os06g04270.1 | transketolase, chloroplast precursor, putative, expressed | 1.3.8 PS.calvin cyle.transketolase | | 0.739 | 0.000 |
| **Protein metabolism** | |  |  | |  |  |
| B8AQ38 | Os03g10190.1 | seryl-tRNA synthetase, putative, expressed | 29.1.11 protein.aa activation.serine-tRNA ligase | | 0.668 | 0.000 |
| B8AGR5 | Os02g46130.1 | aspartyl-tRNA synthetase, putative, expressed | 29.1.12 protein.aa activation.aspartate-tRNA ligase | | 0.482 | 0.000 |
| B8ATF5 | Os04g32650.1 | glycyl-tRNA synthetase 1, mitochondrial precursor, putative, expressed | 29.1.14 protein.aa activation.glycine-tRNA ligase | | 0.494 | 0.001 |
| A2YXJ3 | Os08g42560.1 | glycyl-tRNA synthetase 1, mitochondrial precursor, putative, expressed | 29.1.14 protein.aa activation.glycine-tRNA ligase | | 0.515 | 0.002 |
| B8ADM6 | Os01g09000.1 | glutaminyl-tRNA synthetase, putative, expressed | 29.1.18 protein.aa activation.glutamine-tRNA ligase | | 0.600 | 0.002 |
| C8TFD5 | Os08g19850.1 | threonyl-tRNA synthetase, mitochondrial precursor, putative, expressed | 29.1.3 protein.aa activation.threonine-tRNA ligase | | 0.454 | 0.000 |
| A2Z0N8 | Os09g21110.1 | leucyl-tRNA synthetase, cytoplasmic, putative, expressed | 29.1.4 protein.aa activation. leucine-tRNA ligase | | 0.624 | 0.012 |
| B8BPH6 | Os12g25710.3 | bifunctional aminoacyl-tRNA synthetase, putative, expressed | 29.1.40 protein.aa activation.bifunctional aminoacyl-tRNA synthetase | | 0.663 | 0.000 |
| B8BFZ6 | Os10g10244.1 | alanyl-tRNA synthetase, mitochondrial precursor, putative, expressed | 29.1.7 protein.aa activation.alanine-tRNA ligase | | 0.684 | 0.000 |
| B8APY8 | Os03g48850.2 | valyl-tRNA synthetase, putative, expressed | 29.1.9 protein.aa activation. valine-tRNA ligase | | 0.550 | 0.007 |
| A2ZLS7 | Os12g38000.1 | 60S ribosomal protein L2, putative, expressed | 29.2.1.1 protein.synthesis.ribosomal protein.prokaryotic | | 0.636 | 0.000 |
| A2WLK9 | Os01g09510.4 | 60S acidic ribosomal protein, putative, expressed | 29.2.2 protein.synthesis.misc ribososomal protein | | 1.530 | 0.000 |
| B8A7P8 | Os01g67134.1 | 60S ribosomal protein L5-1, putative, expressed | 29.2.2 protein.synthesis.misc ribososomal protein | | 0.696 | 0.001 |
| A2WZV1 | Os02g01560.1 | 40S ribosomal protein S4, putative, expressed | 29.2.2 protein.synthesis.misc ribososomal protein | | 0.707 | 0.000 |
| B8AI71 | Os02g06700.2 | 40S ribosomal protein S14, putative, expressed | 29.2.2 protein.synthesis.misc ribososomal protein | | 0.738 | 0.000 |
| A2X3J5 | Os02g18550.1 | 40S ribosomal protein S3a, putative, expressed | 29.2.2 protein.synthesis.misc ribososomal protein | | 1.712 | 0.000 |
| A2X6N1 | Os02g37862.1 | 60S ribosomal protein L6, putative, expressed | 29.2.2 protein.synthesis.misc ribososomal protein | | 0.691 | 0.000 |
| A2XDL4 | Os03g10340.1 | 40S ribosomal protein S3a, putative, expressed | 29.2.2 protein.synthesis.misc ribososomal protein | | 1.665 | 0.000 |
| B8AK27 | Os03g14530.1 | 40S ribosomal protein S20, putative, expressed | 29.2.2 protein.synthesis.misc ribososomal protein | | 0.491 | 0.005 |
| B8APD9 | Os03g22180.1 | 60S ribosomal protein L18, putative, expressed | 29.2.2 protein.synthesis.misc ribososomal protein | | 1.703 | 0.003 |
| A2XIT7 | Os03g38000.1 | 40S ribosomal protein S3, putative, expressed | 29.2.2 protein.synthesis.misc ribososomal protein | | 0.690 | 0.000 |
| A2XS31 | Os04g27860.1 | 40S ribosomal protein S27, putative, expressed | 29.2.2 protein.synthesis.misc ribososomal protein | | 0.705 | 0.000 |
| A2XS59 | Os04g28180.1 | 40S ribosomal protein S8, putative, expressed | 29.2.2 protein.synthesis.misc ribososomal protein | | 0.524 | 0.000 |
| B8ARB0 | Os04g42270.2 | 60S ribosomal protein L23a, putative, expressed | 29.2.2 protein.synthesis.misc ribososomal protein | | 0.638 | 0.000 |
| A2XX38 | Os04g50990.1 | 60S ribosomal protein L12, putative, expressed | 29.2.2 protein.synthesis.misc ribososomal protein | | 2.217 | 0.000 |
| B8ATU3 | Os04g51630.2 | 60S ribosomal protein L7-2, putative, expressed | 29.2.2 protein.synthesis.misc ribososomal protein | | 1.379 | 0.000 |
| A2Y0K0 | Os05g06310.1 | 60S ribosomal protein L18, putative, expressed | 29.2.2 protein.synthesis.misc ribososomal protein | | 0.737 | 0.000 |
| B8AZ52 | Os05g11710.1 | 60S ribosomal protein L11, putative, expressed | 29.2.2 protein.synthesis.misc ribososomal protein | | 0.445 | 0.000 |
| B8AW24 | Os05g19370.1 | 60S ribosomal protein L15, putative, expressed | 29.2.2 protein.synthesis.misc ribososomal protein | | 0.725 | 0.000 |
| A2Y3W4 | Os05g30530.1 | 40S ribosomal protein S4, putative, expressed | 29.2.2 protein.synthesis.misc ribososomal protein | | 0.586 | 0.018 |
| A2Y7B5 | Os05g48220.2 | 60S ribosomal protein L33-B, putative, expressed | 29.2.2 protein.synthesis.misc ribososomal protein | | 0.394 | 0.000 |
| A2Y7B6 | Os05g48320.1 | 60S ribosomal protein L37a, putative, expressed | 29.2.2 protein.synthesis.misc ribososomal protein | | 0.661 | 0.006 |
| A2Y7H9 | Os05g49030.1 | 60S ribosomal protein L18a, putative, expressed | 29.2.2 protein.synthesis.misc ribososomal protein | | 0.737 | 0.000 |
| A2Y8K1 | Os06g02510.3 | 60S ribosomal protein L13-2, putative, expressed | 29.2.2 protein.synthesis.misc ribososomal protein | | 0.754 | 0.004 |
| B8B3L8 | Os06g36160.1 | 40S ribosomal protein S24, putative, expressed | 29.2.2 protein.synthesis.misc ribososomal protein | | 0.799 | 0.012 |
| A2YJM2 | Os07g05580.1 | 40S ribosomal protein S12, putative, expressed | 29.2.2 protein.synthesis.misc ribososomal protein | | 1.230 | 0.004 |
| A2YIS2 | Os07g08330.1 | 60S ribosomal protein L4, putative, expressed | 29.2.2 protein.synthesis.misc ribososomal protein | | 1.889 | 0.000 |
| A2YJC1 | Os07g10720.2 | 40S ribosomal protein S15a, putative, expressed | 29.2.2 protein.synthesis.misc ribososomal protein | | 0.644 | 0.000 |
| A2YNT9 | Os07g42950.1 | 40S ribosomal protein S6, putative, expressed | 29.2.2 protein.synthesis.misc ribososomal protein | | 0.698 | 0.000 |
| A2YPV2 | Os07g47710.1 | 60S ribosomal protein L22-2, putative, expressed | 29.2.2 protein.synthesis.misc ribososomal protein | | 0.685 | 0.014 |
| A2YQN3 | Os08g02340.2 | 60S acidic ribosomal protein P1, putative, expressed | 29.2.2 protein.synthesis.misc ribososomal protein | | 1.381 | 0.000 |
| A6N1T9 | Os08g03640.1 | 60S acidic ribosomal protein P0, putative, expressed | 29.2.2 protein.synthesis.misc ribososomal protein | | 2.797 | 0.000 |
| A2Z2J3 | Os09g31180.1 | 60S ribosomal protein L9, putative, expressed | 29.2.2 protein.synthesis.misc ribososomal protein | | 0.188 | 0.000 |
| Q3MST7 | Os09g32532.2 | 60S ribosomal protein L32, putative, expressed | 29.2.2 protein.synthesis.misc ribososomal protein | | 0.675 | 0.008 |
| A2Z2X9 | Os09g32976.1 | 60S ribosomal protein L7a, putative, expressed | 29.2.2 protein.synthesis.misc ribososomal protein | | 1.562 | 0.000 |
| A2Z764 | Os10g27190.1 | 40S ribosomal protein S17-4, putative, expressed | 29.2.2 protein.synthesis.misc ribososomal protein | | 0.786 | 0.007 |
| B8BKA7 | Os11g24610.1 | 60S ribosomal protein L38, putative, expressed | 29.2.2 protein.synthesis.misc ribososomal protein | | 0.481 | 0.014 |
| A2ZFU3 | Os11g38959.1 | 40S ribosomal protein S9, putative, expressed | 29.2.2 protein.synthesis.misc ribososomal protein | | 0.782 | 0.000 |
| A2ZHU8 | Os12g03880.1 | 60S acidic ribosomal protein P0, putative, expressed | 29.2.2 protein.synthesis.misc ribososomal protein | | 0.720 | 0.000 |
| A2ZIF8 | Os12g07010.1 | 60S ribosomal protein L3, putative, expressed | 29.2.2 protein.synthesis.misc ribososomal protein | | 0.705 | 0.022 |
| A2XIU7 | Os03g38260.1 | 60S ribosomal protein L19-3, putative, expressed | 29.2.2 protein.synthesis.misc ribososomal protein; 34.15 transport.potassium | | 0.751 | 0.001 |
| A4Q8X0 | Os01g73880.1 | eukaryotic translation initiation factor 4E-1, putative, expressed | 29.2.3 protein.synthesis.initiation | | 0.451 | 0.043 |
| A2XD90 | Os03g08450.1 | eukaryotic translation initiation factor 3 subunit 12, putative, expressed | 29.2.3 protein.synthesis.initiation | | 0.429 | 0.001 |
| A2Y7J4 | Os05g49150.1 | eukaryotic translation initiation factor 3 subunit 7, putative, expressed | 29.2.3 protein.synthesis.initiation | | 0.625 | 0.036 |
| B8B6V6 | Os07g03230.2 | eukaryotic translation initiation factor 3 subunit 8, putative, expressed | 29.2.3 protein.synthesis.initiation | | 0.714 | 0.046 |
| A2YZW8 | Os09g15770.1 | eukaryotic translation initiation factor 5, putative, expressed | 29.2.3 protein.synthesis.initiation | | 0.372 | 0.017 |
| B8BIC4 | Os10g41960.1 | eukaryotic translation initiation factor 3 subunit 9, putative, expressed | 29.2.3 protein.synthesis.initiation | | 0.631 | 0.006 |
| B8AE83 | Os02g12800.2 | elongation factor 1-gamma 1, putative, expressed | 29.2.4 protein.synthesis.elongation | | 0.744 | 0.007 |
| B8AEQ9 | Os02g38210.1 | elongation factor Tu, chloroplast precursor, putative, expressed | 29.2.4 protein.synthesis.elongation | | 0.644 | 0.000 |
| A2Y3Z4 | Os05g31000.1 | nascent polypeptide-associated complex alpha subunit-like protein, putative, expressed | 29.2.4 protein.synthesis.elongation | | 0.682 | 0.000 |
| B8B3U4 | Os06g37440.1 | elongation factor 1-gamma 3, putative, expressed | 29.2.4 protein.synthesis.elongation | | 0.766 | 0.006 |
| A2Y3K8 | Os05g28510.4 | importin beta-1 subunit, putative, expressed | 29.3.1 protein.targeting.nucleus | | 0.614 | 0.043 |
| A2YXF8 | Os08g42000.1 | nuclear transport factor 2, putative, expressed | 29.3.1 protein.targeting.nucleus | | 0.316 | 0.000 |
| A2ZDV4 | Os11g24560.1 | protein transport protein Sec23A, putative, expressed | 29.3.4.2 protein.targeting.secretory pathway.golgi | | 0.508 | 0.002 |
| A2WXS7 | Os01g66560.1 | signal recognition particle 72 kDa protein, putative, expressed | 29.3.4.99 protein.targeting.secretory pathway.unspecified | | 0.550 | 0.009 |
| A2XN99 | Os03g59740.3 | ADP-ribosylation factor, putative, expressed | 29.3.4.99 protein.targeting.secretory pathway.unspecified | | 0.517 | 0.000 |
| B8ANE8 | Os03g20630.2 | zinc-binding protein, putative, expressed | 29.4 protein.postranslational modification | | 0.649 | 0.000 |
| B8BD97 | Os09g07510.2 | serine/threonine-protein phosphatase 2A 65 kDa regulatory subunit Abeta isoform, putative, expressed | 29.4 protein.postranslational modification | | 0.616 | 0.000 |
| A2X2G9 | Os02g12650.1 | puromycin-sensitive aminopeptidase, putative, expressed | 29.5 protein.degradation | | 0.396 | 0.000 |
| B8AGD0 | Os02g44520.1 | tripeptidyl-peptidase 2, putative, expressed | 29.5.1 protein.degradation.subtilases | | 0.671 | 0.005 |
| B8AHG2 | Os02g04100.1 | proteasome subunit alpha type 1, putative, expressed | 29.5.11.20 protein.degradation.ubiquitin.proteasom | | 0.402 | 0.018 |
| B8AGL8 | Os02g21970.1 | 26S protease regulatory subunit 6B, putative, expressed | 29.5.11.20 protein.degradation.ubiquitin.proteasom | | 2.493 | 0.047 |
| B8AFT1 | Os02g42320.2 | proteasome subunit alpha type 2, putative, expressed | 29.5.11.20 protein.degradation.ubiquitin.proteasom | | 0.776 | 0.001 |
| A2XD75 | Os03g08280.2 | proteasome subunit alpha type 6, putative, expressed | 29.5.11.20 protein.degradation.ubiquitin.proteasom | | 0.555 | 0.001 |
| A2XKY8 | Os03g48930.1 | proteasome subunit beta type 2, putative, expressed | 29.5.11.20 protein.degradation.ubiquitin.proteasom | | 0.486 | 0.000 |
| A2XZF9 | Os05g01450.1 | eukaryotic translation initiation factor 3 subunit 5, putative, expressed | 29.5.11.20 protein.degradation.ubiquitin.proteasom | | 0.621 | 0.000 |
| A2Y3X5 | Os05g30800.1 | 26S proteasome non-ATPase regulatory subunit 14, putative, expressed | 29.5.11.20 protein.degradation.ubiquitin.proteasom | | 0.398 | 0.017 |
| A2YSI1 | Os08g12820.1 | 26S proteasome non-ATPase regulatory subunit 1, putative, expressed | 29.5.11.20 protein.degradation.ubiquitin.proteasom | | 0.556 | 0.001 |
| B8BEG9 | Os09g15750.1 | 26S proteasome non-ATPase regulatory subunit 2, expressed | 29.5.11.20 protein.degradation.ubiquitin.proteasom | | 0.452 | 0.000 |
| B8A797 | Os01g48280.1 | ubiquitin-conjugating enzyme E2 N, putative, expressed | 29.5.11.3 protein.degradation.ubiquitin.E2 | | 0.265 | 0.013 |
| A2XYQ9 | Os04g57220.3 | ubiquitin-conjugating enzyme E2-17 kDa, putative, expressed | 29.5.11.3 protein.degradation.ubiquitin.E2 | | 0.690 | 0.001 |
| A2Y5G0 | Os05g38550.2 | ubiquitin-conjugating enzyme E2 7, putative, expressed | 29.5.11.3 protein.degradation.ubiquitin.E2 | | 1.350 | 0.000 |
| A2ZDZ0 | Os11g26910.1 | SKP1-like protein 1A, putative, expressed | 29.5.11.4.3.1 protein.degradation.ubiquitin.E3.SCF.SKP | | 0.290 | 0.002 |
| A2XYT1 | Os04g57440.1 | cysteine protease 1 precursor, putative, expressed | 29.5.3 protein.degradation.cysteine protease | | 0.528 | 0.002 |
| B8A762 | Os01g47410.2 | aspartic proteinase oryzasin-1 precursor, putative, expressed | 29.5.4 protein.degradation.aspartate protease | | 1.489 | 0.000 |
| B8AM77 | Os03g41419.1 | serpin family protein, expressed | 29.5.5 protein.degradation.serine protease | | 0.623 | 0.018 |
| B8BNQ4 | Os12g12850.2 | ATP-dependent Clp protease ATP-binding subunit clpA homolog,chloroplast precursor, putative, expressed | 29.5.5 protein.degradation.serine protease | | 0.624 | 0.004 |
| B8BH45 | Os10g30580.1 | cell division control protein 48 homolog E, putative, expressed | 29.5.9 protein.degradation.AAA type; 29.5.11.20 protein.degradation.ubiquitin.proteasom | | 0.654 | 0.004 |
| A2XPB4 | Os03g64210.1 | T-complex protein, putative, expressed | 29.6 protein.folding | | 0.776 | 0.001 |
| B8ASU6 | Os04g46620.1 | T-complex protein 1 subunit alpha, putative, expressed | 29.6 protein.folding | | 2.151 | 0.000 |
| B8B3P0 | Os06g09679.2 | chaperonin, chloroplast precursor, putative, expressed | 29.6 protein.folding | | 0.683 | 0.000 |
| B8B9V2 | Os08g25090.2 | co-chaperone GrpE protein, putative, expressed | 29.6 protein.folding | | 0.615 | 0.006 |
| B8BHB9 | Os10g32550.1 | chaperonin CPN60-1, mitochondrial precursor, putative, expressed | 29.6 protein.folding | | 3.213 | 0.043 |
| **Redox** |  |  |  | |  |  |
| B8ALD1 | Os03g58630.1 | thioredoxin H-type 5, putative, expressed | 21.1 redox.thioredoxin | | 0.673 | 0.000 |
| B8AYG0 | Os05g06430.2 | OsPDIL2-1 - Oryza sativa protein disulfide isomerase, expressed | 21.1 redox.thioredoxin | | 0.642 | 0.004 |
| B8B6B6 | Os07g49400.2 | OsAPx2 - Cytosolic Ascorbate Peroxidase encoding gene, expressed | 21.2.1 redox.ascorbate and glutathione.ascorbate | | 0.201 | 0.006 |
| A2YXU4 | Os08g43560.1 | OsAPx4 - Peroxisomal Ascorbate Peroxidase encoding gene, expressed | 21.2.1 redox.ascorbate and glutathione.ascorbate | | 0.390 | 0.000 |
| B8B5S6 | Os07g27790.1 | glutamate--cysteine ligase, chloroplast precursor, putative, expressed | 21.2.2 redox.ascorbate and glutathione.glutathione | | 0.574 | 0.001 |
| A2WTQ2 | Os01g48420.1 | peroxiredoxin-5, mitochondrial precursor, putative, expressed | 21.5 redox.peroxiredoxin | | 0.514 | 0.003 |
| B8AJ36 | Os02g33450.2 | 2-cys peroxiredoxin BAS1, chloroplast precursor, putative, expressed | 21.5 redox.peroxiredoxin | | 0.664 | 0.000 |
| A2YF78 | Os06g42000.1 | peroxiredoxin-5, mitochondrial precursor, putative, expressed | 21.5 redox.peroxiredoxin | | 0.550 | 0.012 |
| B8AWM4 | Os05g25850.1 | superoxide dismutase, mitochondrial precursor, putative, expressed | 21.6 redox.dismutases and catalases | | 0.701 | 0.000 |
| B8B2L5 | Os06g51150.1 | catalase isozyme B, putative, expressed | 21.6 redox.dismutases and catalases | | 1.655 | 0.000 |
| **RNA** |  |  |  | |  |  |
| C8TFM3 | Os08g22354.1 | polyadenylate-binding protein 2, putative, expressed | 27.1 RNA.processing | | 0.456 | 0.001 |
| A2YYH2 | Os09g02700.1 | polyadenylate-binding protein 2, putative, expressed | 27.1 RNA.processing | | 1.358 | 0.000 |
| A2Y4P5 | Os05g34310.1 | NAC domain-containing protein 94, putative, expressed | 27.3.27 RNA.regulation of transcription.NAC domain transcription factor family | | 2.030 | 0.000 |
| B8AXI7 | Os05g51830.1 | histone deacetylase 2b, putative, expressed | 27.3.55 RNA.regulation of transcription.HDA | | 0.583 | 0.002 |
| B8APG2 | Os03g22740.1 | nucleolar protein NOP5, putative, expressed | 27.3.67 RNA.regulation of transcription.putative transcription regulator | | 0.636 | 0.042 |
| B8BHC8 | Os05g28280.1 | proliferation-associated protein 2G4, putative, expressed | 27.3.67 RNA.regulation of transcription.putative transcription regulator | | 0.723 | 0.000 |
| A2WS70 | Os01g41550.1 | aspartic proteinase nepenthesin-2 precursor, putative, expressed | 27.3.99 RNA.regulation of transcription.unclassified | | 0.525 | 0.001 |
| A2WYB5 | Os01g68790.3 | RNA recognition motif containing protein, putative, expressed | 27.4 RNA | | 0.659 | 0.000 |
| B8AF48 | Os02g57640.1 | RNA-binding protein Nova-1, putative, expressed | 27.4 RNA.RNA binding | | 0.696 | 0.001 |
| B8B7X7 | Os07g08960.1 | glycine-rich RNA-binding protein 7, putative, expressed | 27.4 RNA.RNA binding | | 0.176 | 0.009 |
| B8BEL6 | Os09g39180.1 | ribonucleoprotein, chloroplast precursor, putative, expressed | 27.4 RNA.RNA binding | | 0.489 | 0.007 |
| A2ZKT5 | Os12g31800.1 | glycine-rich RNA-binding protein 7, putative, expressed | 27.4 RNA.RNA binding | | 0.672 | 0.001 |
| **Secondary metabolism** | |  |  | |  |  |
| A2ZMQ9 | Os12g42090.2 | inner envelope membrane protein, chloroplast precursor, putative, expressed | 16.1.3.3 secondary metabolism.isoprenoids.tocopherol biosynthesis.MPBQ/MSBQ methyltransferase | | 0.534 | 0.000 |
| A2YY66 | Os08g44840.1 | acyltransferase, putative, expressed | 16.2 secondary metabolism.phenylpropanoids | | 0.297 | 0.020 |
| A6N176 | Os02g41630.2 | phenylalanine ammonia-lyase, putative, expressed | 16.2.1.1 secondary metabolism.phenylpropanoids.lignin biosynthesis.PAL | | 0.202 | 0.032 |
| A2Z2F5 | Os09g30360.1 | caffeoyl-CoA O-methyltransferase 1, putative, expressed | 16.2.1.6 secondary metabolism.phenylpropanoids.lignin biosynthesis.CCoAOMT | | 0.455 | 0.027 |
| A2YRE2 | Os08g06100.1 | quercetin 3-O-methyltransferase 1, putative, expressed | 16.2.1.9 secondary metabolism.phenylpropanoids.lignin biosynthesis.COMT | | 0.798 | 0.000 |
| B8AV58 | Os04g39020.1 | betaine-aldehyde dehydrogenase, putative, expressed | 16.4.2 secondary metabolism.N misc.betaine | | 0.506 | 0.001 |
| **Signalling** | |  |  | |  |  |
| B8B1A4 | Os06g01500.1 | IQ calmodulin-binding motif family protein, expressed | 30.3 signalling.calcium | | 1.810 | 0.025 |
| B8B4R4 | Os07g14270.3 | calreticulin precursor, putative, expressed | 30.3 signalling.calcium | | 0.792 | 0.002 |
| B8ABU1 | Os01g15010.1 | GTP-binding protein SAR1A, putative, expressed | 30.5 signalling.G-proteins | | 0.565 | 0.001 |
| A2WRD4 | Os01g37800.1 | ras-related protein RIC1, putative, expressed | 30.5 signalling.G-proteins | | 0.786 | 0.000 |
| A2WTW6 | Os01g49290.1 | WD repeat-containing protein, putative, expressed | 30.5 signalling.G-proteins | | 0.690 | 0.000 |
| B8ABA2 | Os01g59980.1 | zinc finger family protein, putative, expressed | 30.5 signalling.G-proteins | | 0.758 | 0.003 |
| A2XN85 | Os03g59590.1 | ATP/GTP/Ca++ binding protein, putative, expressed | 30.5 signalling.G-proteins | | 0.697 | 0.000 |
| A2Y4R1 | Os05g34540.1 | rab GDP dissociation inhibitor alpha, putative, expressed | 30.5 signalling.G-proteins | | 1.668 | 0.024 |
| B8B4C1 | Os06g39875.1 | ras-related protein, putative, expressed | 30.5 signalling.G-proteins | | 0.637 | 0.010 |
| B8B4B7 | Os06g39760.1 | serine-threonine kinase receptor-associated protein, putative, expressed | 30.5 signalling.G-proteins; 33.99 development.unspecified | | 0.655 | 0.000 |
| A2XL95 | Os03g50290.3 | 14-3-3-like protein S94, putative, expressed | 30.7 signalling.14-3-3 proteins | | 0.390 | 0.000 |
| A2XUA6 | Os04g38870.5 | 14-3-3-like protein GF14-6, putative, expressed | 30.7 signalling.14-3-3 proteins | | 0.707 | 0.000 |
| **Stress** |  |  |  | |  |  |
| B8AMD8 | Os03g03810.1 | flower-specific gamma-thionin precursor, putative, expressed | 20.1 stress.biotic | | 1.246 | 0.008 |
| A2Y2C4 | Os05g15770.1 | xylanase inhibitor protein 2 precursor, putative, expressed | 20.1 stress.biotic | | 1.971 | 0.005 |
| A2YJG1 | Os07g11380.1 | seed allergenic protein RAG2 precursor, putative, expressed | 20.1 stress.biotic | | 1.449 | 0.000 |
| A2YJG2 | Os07g11410.1 | seed allergenic protein RAG2 precursor, putative, expressed | 20.1 stress.biotic | | 1.290 | 0.000 |
| A2YNL0 | Os07g41810.4 | stress responsive A/B Barrel domain containing protein, expressed | 20.1 stress.biotic | | 1.530 | 0.020 |
| A2ZGZ0 | Os11g47520.1 | xylanase inhibitor protein 2 precursor, putative, expressed | 20.1 stress.biotic | | 1.679 | 0.035 |
| A2WLE4 | Os01g08560.2 | heat shock 70 kDa protein 4, putative, expressed | 20.2.1 stress.abiotic.heat | | 0.678 | 0.000 |
| B8AG03 | Os02g43020.1 | heat shock protein STI, putative, expressed | 20.2.1 stress.abiotic.heat | | 0.747 | 0.001 |
| A2X8U7 | Os02g48110.1 | oxygen-regulated protein precursor, putative, expressed | 20.2.1 stress.abiotic.heat | | 0.629 | 0.013 |
| A2XF40 | Os03g16860.1 | heat shock cognate 70 kDa protein 2, putative, expressed | 20.2.1 stress.abiotic.heat | | 0.701 | 0.001 |
| A2XMP7 | Os03g57340.1 | dnaJ protein, putative, expressed | 20.2.1 stress.abiotic.heat | | 0.711 | 0.000 |
| A2YH08 | Os06g50300.1 | heat shock protein, putative, expressed | 20.2.1 stress.abiotic.heat | | 0.672 | 0.000 |
| B8BCQ3 | Os09g29840.1 | endoplasmin precursor, putative, expressed | 20.2.1 stress.abiotic.heat | | 0.763 | 0.000 |
| A2Z2G1 | Os09g30412.1 | heat shock protein 81-3, putative, expressed | 20.2.1 stress.abiotic.heat | | 0.634 | 0.000 |
| B8AGK8 | Os02g02870.1 | glycine-rich protein 2, putative, expressed | 20.2.2 stress.abiotic.cold | | 0.756 | 0.045 |
| A2X3I3 | Os02g18410.1 | DREPP2 protein, putative, expressed | 20.2.3 stress.abiotic.drought/salt | | 0.736 | 0.037 |
| A2XUF3 | Os04g39150.1 | major latex protein 22, putative, expressed | 20.2.99 stress.abiotic.unspecified | | 0.515 | 0.000 |
| A2Y689 | Os05g42230.1 | ER6 protein, putative, expressed | 20.2.99 stress.abiotic.unspecified | | 1.998 | 0.010 |
| **TCA/org** |  |  |  | |  |  |
| A2XPT6 | Os04g02900.1 | pyruvate dehydrogenase E1 component alpha subunit, putative, expressed | 8.1.1.1 TCA / org. transformation.TCA.pyruvate DH.E1 | | 0.598 | 0.001 |
| A2Z2Z0 | Os09g33500.1 | pyruvate dehydrogenase E1 component subunit beta, mitochondrial precursor, putative, expressed | 8.1.1.1 TCA / org. transformation.TCA.pyruvate DH.E1 | | 0.562 | 0.000 |
| B8BB05 | Os08g33440.1 | dihydrolipoyllysine-residue acetyltransferase component of pyruvatedehydrogenase complex, putative, expressed | 8.1.1.2 TCA / org. transformation.TCA.pyruvate DH.E2 | | 0.624 | 0.000 |
| A2WTC5 | Os01g46610.1 | isocitrate dehydrogenase, chloroplast precursor, putative, expressed | 8.1.4 TCA / org. transformation.TCA.IDH | | 0.295 | 0.000 |
| A2X7C5 | Os02g40830.1 | succinyl-CoA ligase beta-chain, mitochondrial precursor, putative, expressed | 8.1.6 TCA / org. transformation.TCA.succinyl-CoA ligase | | 0.578 | 0.000 |
| B8B729 | Os07g04240.1 | succinate dehydrogenase flavoprotein subunit,mitochondrial precursor, putative, expressed | 8.1.7 TCA / org. transformation.TCA.succinate dehydrogenase | | 0.725 | 0.042 |
| B8AP09 | Os03g21950.1 | fumarate hydratase 1, mitochondrial precursor, putative, expressed | 8.1.8 TCA / org. transformation.TCA.fumarase | | 0.642 | 0.002 |
| A2WNV6 | Os01g19450.1 | ATP-citrate synthase subunit 1, putative, expressed | 8.2.11 TCA / org. transformation.other organic acid transformaitons.atp-citrate lyase | | 0.689 | 0.008 |
| **Tetrapyrrole metabolism** | | |  | |  |  |
| B8B936 | Os08g41990.1 | glutamate-1-semialdehyde 2,1-aminomutase, chloroplast precursor, putative, expressed | 19.3 tetrapyrrole synthesis.GSA | | 0.454 | 0.038 |
| **Transporter** | |  |  | |  |  |
| A2WRY9 | Os01g40570.1 | outer plastidial membrane protein porin, putative, expressed | 34.2 transporter.sugars | | 0.592 | 0.001 |
| B8AQH5 | Os03g10510.1 | outer mitochondrial membrane protein porin, putative, expressed | 34.2 transporter.sugars | | 0.504 | 0.000 |
| A2Z0D1 | Os09g19734.3 | isochorismate synthase 1, chloroplast precursor, putative, expressed | 34.2 transporter.sugars | | 0.688 | 0.000 |
| W8QQX9 | Os05g06480.1 | pyrophosphate-energized vacuolar membrane proton pump, putative, expressed | 34.3 transport.amino acids | | 0.512 | 0.000 |
| B8BBE6 | Os08g08840.2 | glucose-6-phosphate/phosphate translocator 2, chloroplast precursor, putative, expressed | 34.8 transport.metabolite transporters at the envelope membrane | | 1.325 | 0.000 |
| B8AJ45 | Os02g52860.1 | phosphate carrier protein, mitochondrial precursor, putative, expressed | 34.9 transport.metabolite transporters at the mitochondrial membrane | | 0.292 | 0.015 |
| A2Y1H8 | Os05g11780.1 | mitochondrial 2-oxoglutarate/malate carrier protein, putative, expressed | 34.9 transport.metabolite transporters at the mitochondrial membrane | | 0.440 | 0.002 |
| A2YE72 | Os06g37180.1 | vacuolar ATP synthase subunit B isoform 1, putative, expressed | 34.1 transport.p- and v-ATPases | | 0.441 | 0.000 |
