# Supplementary figures and images for: SWATH-MS Quantitative Analysis of Proteins in the Rice Inferior and Superior Spikelets during Grain Filling
Source: Front Plant Sci. 2016 Dec 20;7:1926. doi: 10.3389/fpls.2016.01926 (PMC5169098; doi:10.3389/fpls.2016.01926)

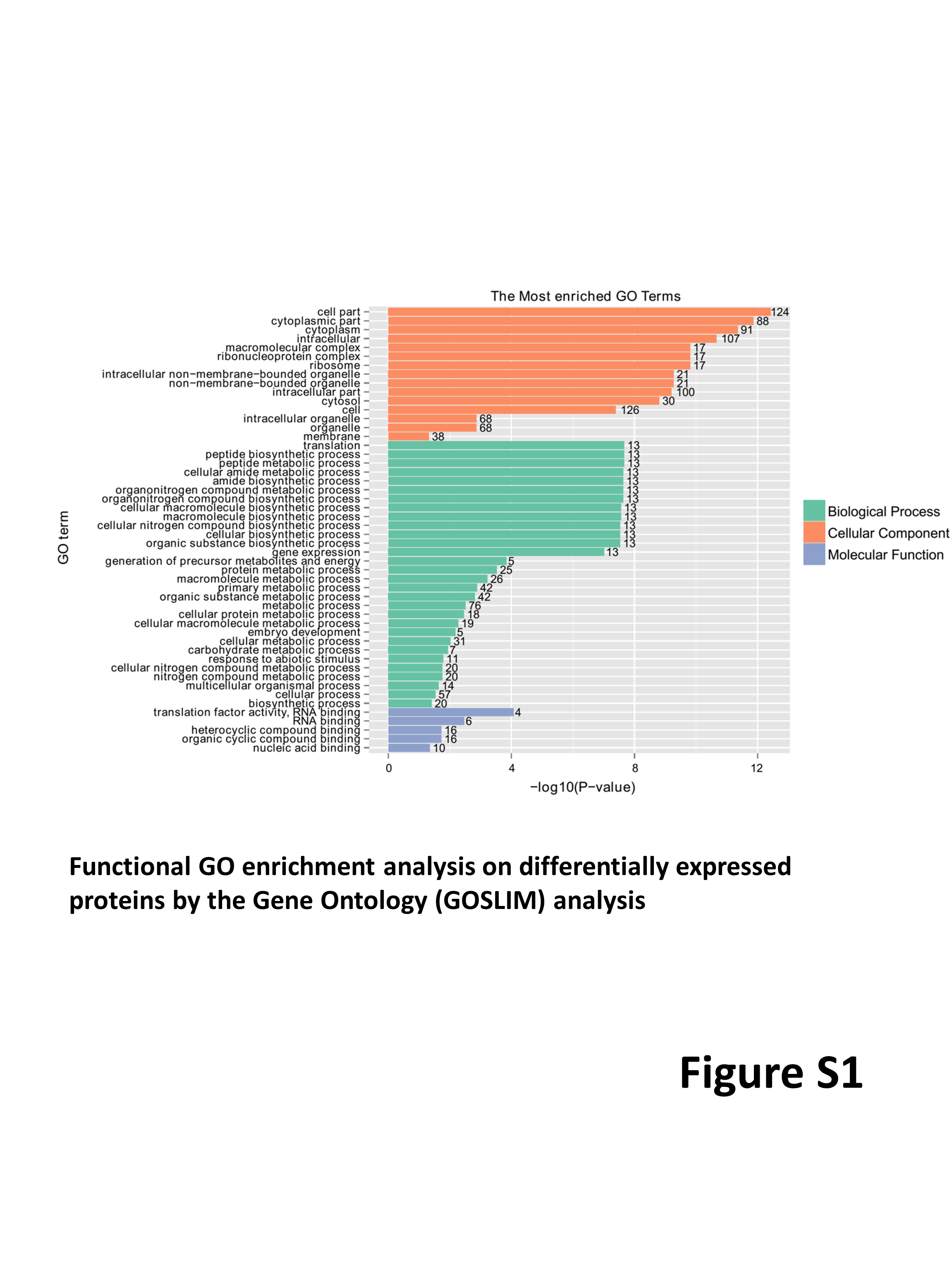

Supplement: Supplementary file 4 [file Image_1.TIF]
